# HuR/Cx40 downregulation causes coronary microvascular dysfunction in type 2 diabetes

**DOI:** 10.1172/jci.insight.147982

**Published:** 2021-11-08

**Authors:** Rui Si, Jody Tori O. Cabrera, Atsumi Tsuji-Hosokawa, Rui Guo, Makiko Watanabe, Lei Gao, Yun Sok Lee, Jae-Su Moon, Brian T. Scott, Jian Wang, Anthony W. Ashton, Jaladanki N. Rao, Jian-Ying Wang, Jason X.-J. Yuan, Ayako Makino

**Affiliations:** 1 Department of Physiology, The University of Arizona (UA), Tucson, Arizona, USA.; 2 Department of Cardiology, Xijing Hospital, Fourth Military Medical University, Shaanxi, China.; 3 Department of Medicine, UCSD, La Jolla, California, USA.; 4 State Key Laboratory of Respiratory Disease, Guangzhou Institute of Respiratory Disease, The First Affiliated Hospital of Guangzhou Medical University, Guangzhou, China.; 5 Division of Perinatal Research, Kolling Institute of Medical Research, University of Sydney, New South Wales, Australia.; 6 Department of Surgery, University of Maryland School of Medicine, Baltimore, Maryland, USA.

**Keywords:** Vascular Biology, Cardiovascular disease

## Abstract

Patients with diabetes with coronary microvascular disease (CMD) exhibit higher cardiac mortality than patients without CMD. However, the molecular mechanism by which diabetes promotes CMD is poorly understood. RNA-binding protein human antigen R (HuR) is a key regulator of mRNA stability and translation; therefore, we investigated the role of HuR in the development of CMD in mice with type 2 diabetes. Diabetic mice exhibited decreases in coronary flow velocity reserve (CFVR; a determinant of coronary microvascular function) and capillary density in the left ventricle. HuR levels in cardiac endothelial cells (CECs) were significantly lower in diabetic mice and patients with diabetes than the controls. Endothelial-specific HuR-KO mice also displayed significant reductions in CFVR and capillary density. By examining mRNA levels of 92 genes associated with endothelial function, we found that *HuR*, *Cx40*, and *Nox4* levels were decreased in CECs from diabetic and *HuR*-KO mice compared with control mice. Cx40 expression and HuR binding to *Cx40* mRNA were downregulated in CECs from diabetic mice. *Cx40*-KO mice exhibited decreased CFVR and capillary density, whereas endothelium-specific *Cx40* overexpression increased capillary density and improved CFVR in diabetic mice. These data suggest that decreased HuR contributes to the development of CMD in diabetes through downregulation of gap junction protein Cx40 in CECs.

## Graphical abstract

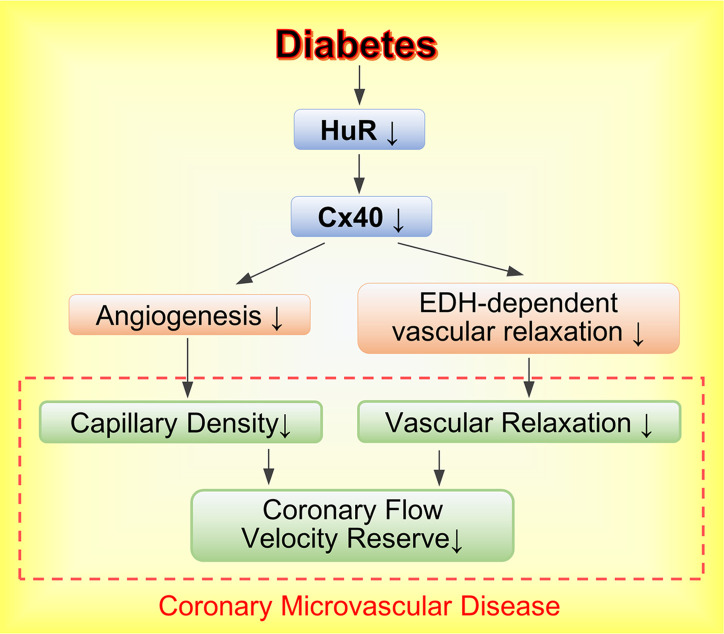

## Introduction

Obstructive coronary artery disease (CAD) is the primary cause of cardiac ischemia and cardiac myocyte (CM) death; however, there is increasing evidence showing that nonobstructive CAD (also known as coronary microvascular disease [CMD]) is another risk factor for increased cardiac mortality (1–3). In fact, stable patients who suffer from ischemia with CMD show a higher risk for major adverse cardiovascular events than patients with CAD (4). However, patients with CMD are commonly treated with cardiovascular medication used for obstructive CAD, like antihypertension, angina therapy, or statin therapy (5). Further understanding of the pathogenic mechanisms specifically involved in CMD is, thus, required to develop novel and unique therapeutic approaches for CMD and CMD-associated cardiac ischemia.

CMD is caused by coronary microvascular dysfunction, including microvascular rarefaction, small vascular remodeling, and attenuated vasodilatation in small coronary arteries (CAs) (1, 6–9). Microvascular rarefaction is due to loss of existing capillary networks and attenuated regeneration of new capillaries. We and other investigators show that capillary density in the heart is decreased in diabetic animals and patients, and that patients with diabetes display reduced coronary flow reserve, a key determinant of coronary microvascular function (10–18). In addition, patients with diabetes with CMD show increased cardiac mortality compared with patients without CMD (19, 20). These studies suggest an urgent need to develop alternative treatments for patients with diabetes with CMD. This study was designed to identify critical genes in diabetic cardiac endothelial cells (CECs) with altered expression levels that influence capillary density and ultimately induce CMD.

Human antigen R (HuR), a member of the embryonic lethal abnormal vision (ELAV) protein family, is an RNA-binding protein (RBP) that contains RNA recognition motifs with high affinity to AU-rich elements in the 3′-untranslated regions (3′-UTR) of transcripts (21). Binding of HuR to mRNAs regulates the stability of mature mRNAs and mRNA decay (22); therefore, HuR regulates mRNA expression levels of many genes via posttranscriptional modification directly or indirectly (e.g., by competing 3′-UTR with microRNA). Ubiquitous KO of *HuR* in mice is embryonically lethal due to a placental defect (23). β Cell–specific *HuR*-KO increases reactive oxygen species (ROS) production and induces cell apoptosis (24). *HuR* deletion in the intestinal epithelium leads to abnormal growth of the small intestine (25). Neuron-specific *HuR*-KO mice exhibit motor deficiency phenotypes (26). Smooth muscle–specific *HuR*-KO mice develop hypertension (27). *HuR*-deletion in mouse CMs, however, does not induce any apparent phenotype (28). The HuR level is significantly decreased in the left ventricle (LV) of patients with heart failure (29) and aorta of rats with spontaneous hypertension (30). In contrast, HuR expression is increased in cancer (31), diabetic nephropathy (32), and diabetic retinopathy (33). HuR overexpression increases angiogenesis because HuR stabilizes *VEGF-A* mRNA and modifies angiogenic activity of endothelial cells (ECs) (34). These data indicate that changes in HuR level contribute to the development of many diseases; it is, however, unclear whether HuR contributes to the development of CMD in diabetes.

In this study, we aimed to investigate coronary microvascular function in diabetic mice and EC-specific *HuR*-KO mice, identify the mRNAs with altered expression in CECs in diabetic mice and *HuR*-KO mice, and target those mRNAs to restore CMD in diabetes.

## Results

### Coronary microvascular dysfunction in diabetic mice.

**Figure 1:**
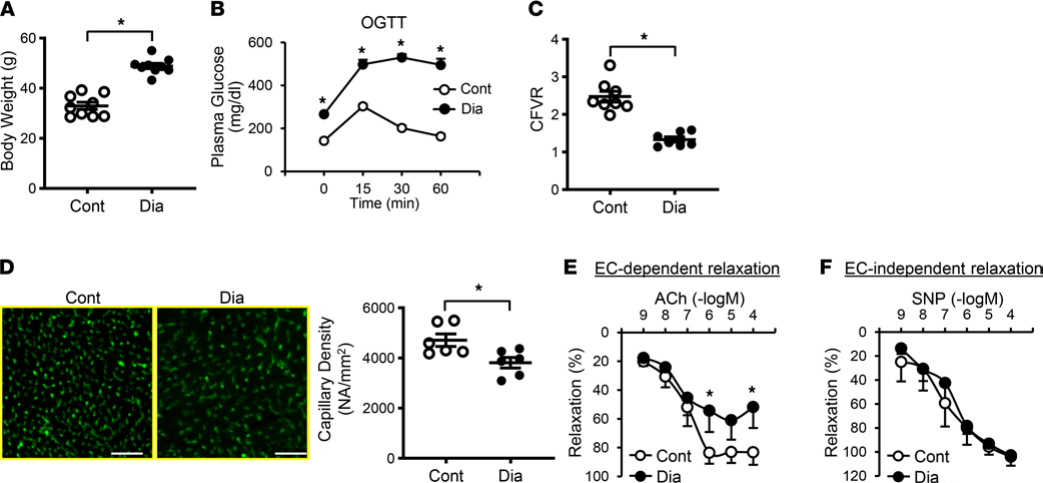
Phenotype of inducible Type 2 diabetic mice. (**A**) Body weight. Control mice (Cont, *n*_mice_ = 9); diabetic mice (Dia, *n*_mice_ = 9). (**B**) Oral glucose-tolerance test (OGTT) 6 hours after fasting. *n*_mice_ = 9 per group. (**C**) Coronary flow velocity reserve (CFVR). Cont, *n*_mice_ = 8; Dia, *n*_mice_ = 7. (**D**) Representative photomicrographs (left) showing capillary density. ECs were stained by BS-lectin-FITC (a marker of ECs, green). Scale bar: 50 μm. Averaged data (right) showing capillary density in control (*n*_mice_ = 6) and diabetic (*n*_mice_ = 6) mice. (**E**) Endothelium-dependent relaxation evaluated by ACh-induced relaxation in coronary arteries (CAs). *n*_mice_ = 8 per group. (**F**) Endothelium-independent relaxation evaluated by SNP-induced relaxation in CAs. *n*_mice_ = 5 per group. Data are presented as mean ± SEM. *P < 0.05 versus Cont. Statistical comparison between time-dependent and dose-dependent curves was made by 2-way ANOVA with Bonferroni post hoc test (**B**, **E**, and **F**). Unpaired Student’s *t* test (2-tailed) was used for comparisons of 2 experimental groups (**A**, **C**, and **D**).

We used a type 2 diabetes (T2D) mouse model generated by high-fat diet feeding and a single low-dose injection of streptozotocin (STZ). This is a well-established mouse model to study T2D, and the metabolic characteristics in this model are very close to those in human T2D associated with Western diet (35–40). Diabetic mice exhibited increased body weight, abnormal glucose tolerance, dyslipidemia, and hyperinsulinemia (Figure 1, Table 1, and Table 2). Coronary flow velocity reserve (CFVR) was measured as a determinant of coronary microvascular function (Supplemental Figure 1; supplemental material available online with this article; https://doi.org/10.1172/jci.insight.147982DS1) (18, 41, 42). Decreased CFVR indicates that mice are suffering from CMD and prone to ischemic heart disease (18). We found that CFVR was significantly reduced in diabetic mice compared with control (Figure 1C). CFVR is regulated by capillary density and small vessel relaxation. In the LV, diabetic mice showed significantly lower capillary density (Figure 1D) and higher EC apoptosis (Supplemental Figure 2) than control mice. We also examined endothelium-dependent relaxation (EDR) in the third and fourth order of CAs by administration of acetylcholine (ACh) in a dose-dependent manner (Supplemental Figures 3 and 4). Figure 1E shows that EDR was significantly attenuated in diabetic mice compared with the control, whereas endothelium-independent relaxation assessed by SNP administration exhibited no difference between control and diabetic CAs (Figure 1F). These results indicate that coronary microvascular function is significantly attenuated in mice with experimental diabetes due potentially to reduced capillary density and attenuated EDR in small CAs.

### Decreased HuR protein level in CECs isolated from diabetic mice and patients.

**Figure 2:**
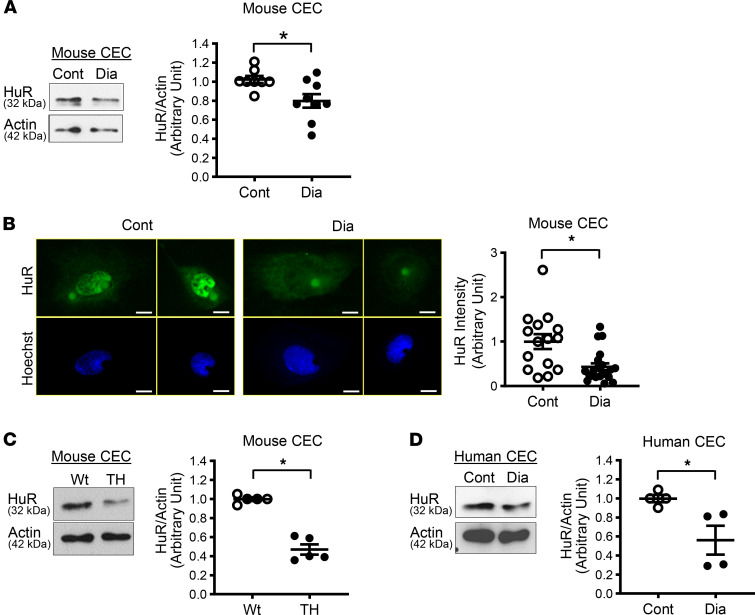
HuR protein levels in cardiac endothelial cells from control and diabetic mice and patients. (**A**) Western blots showing HuR and Actin protein levels in mouse cardiac endothelial cells (CECs) (left panel). The right dot plot shows HuR protein level normalized to Actin. Cont, *n*_mice_ = 8; Dia, *n*_mice_ = 9. *P < 0.05 versus Cont. (**B**) Photomicrographs show typical images of HuR expression in CECs. CECs were stained with HuR (green) and Hoechst (nuclear staining, blue). The right dot plot shows averaged data of HuR intensity. Scale bar: 10 μm. Cont, *n*_cells_ = 15; Dia, *n*_cells_ = 21. Three mice were used per group. *P < 0.05 versus Cont. (**C**) Representative image of Western blots showing HuR and Actin protein levels in CECs from Tallyho mice (TH, spontaneous T2D mice, *n*_mice_=5) and WT mice (*n*_mice_ = 5) (left panel). Dots plot shows summarized data (right panel). *P < 0.05 versus WT. (**D**) HuR protein level in human CECs from control patients (Cont, *n*_patients_ = 4) and patients with diabetes (Dia, *n*_patients_ = 4). *P < 0.05 versus Cont. Data are presented as mean ± SEM. Unpaired Student’s *t* test (2-tailed) was used for comparisons of 2 experimental groups in **A** and **C**. Nonparametric, Mann-Whitney *U* test, was used in **B** and **D**.

Freshly isolated mouse CECs were used to detect HuR levels in control and diabetic mice. HuR levels were significantly decreased in CECs from diabetic mice compared with those from control mice, as determined by Western blot (Figure 2A) and immunofluorescence study (Figure 2B). In line with inducible T2D mouse data, CECs from spontaneous T2D mouse model (TALLYHO/Jng [TH] mice) and CECs from patients with diabetes exhibit a significant decrease in HuR protein level compared with their controls (Figure 2, C and D). On the other hand, HuR levels in cardiac cells (remaining heart cells after depletion of ECs) and aortic SMCs were not different between control and diabetic mice (Supplemental Figure 5). Taken together, HuR protein is selectively downregulated in CECs in diabetic mice, and endothelial downregulation of HuR is a potential contributor to reduced capillary density in the heart and decreased coronary microvascular function in diabetes.

### Endothelium-specific HuR-KO (Tie2-HuR^–/–^) mice develop CMD.

**Figure 3:**
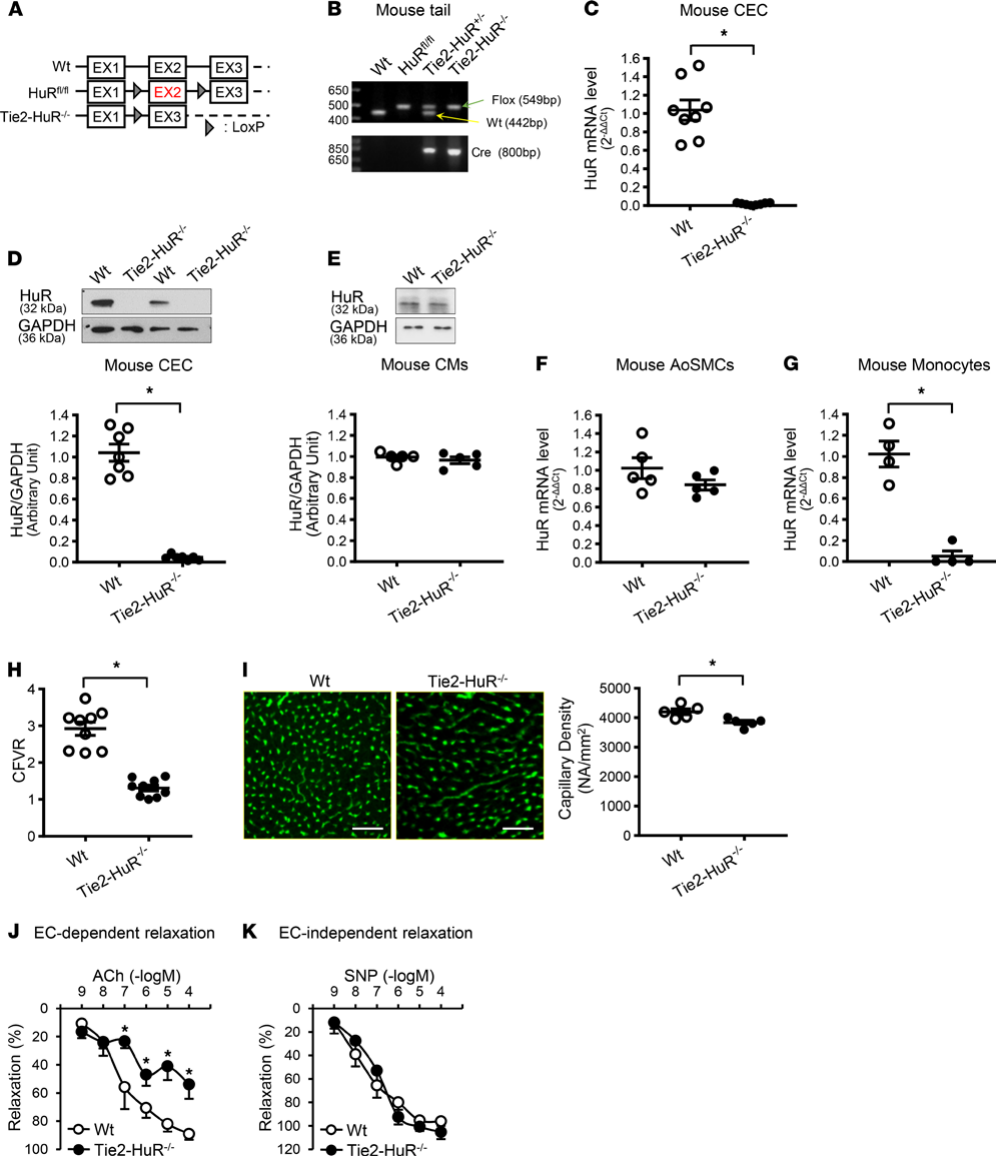
Generation and phenotyping of endothelium-specific HuR–KO mice. (**A**) Diagrams depict the genomic region surrounding exon 2 (EX2) of the WT HuR allele, the HuR-loxP (HuR^fl/fl^) allele with 2 loxP sites (arrowheads) flanking exon 2, and the deleted (Tie2-HuR^–/–^) allele that was produced by crossing HuR^fl/fl^ mice with Tie2-Cre (Tie2-driven Cre–recombinase overexpressing) mice. (**B**) PCR analysis of genomic DNA from tail samples indicates floxed, HuR deletion, and Cre bands in HuR^fl/fl^, Tie2-HuR^+/–^, and Tie2-HuR^–/–^ mice. (**C**) *HuR* mRNA levels in CECs determined by real-time PCR. *n*_mice_ = 8 per group. (**D**) HuR protein levels in CECs determined by Western blot. The bottom dot plot shows HuR protein level normalized to GAPDH. *n*_mice_ = 7 per group. (**E**) HuR protein levels in mouse cardiac myocytes (CMs). *n*_mice_ = 5 per group. (**F**) *HuR* RNA levels in aortic smooth muscle cells (AoSMCs). *n*_mice_ = 5 per group. (**G**) *HuR* RNA levels in monocytes. *n*_mice_ = 4 per group. (**H**) CFVR. *n*_mice_ = 9 per group. (**I**) Representative photomicrographs (left) and summarized data (right) of capillary density. Scale bar: 50 μm. *n*_mice_ = 5 per group. (**J**) Endothelium-dependent relaxation evaluated by ACh-induced relaxation in CAs. WT, *n*_mice_ = 5; Tie2-HuR^–/–^, *n*_mice_ = 6. (**K**) Endothelium-independent relaxation evaluated by SNP-induced relaxation in CAs. WT, *n*_mice_ = 5; Tie2-HuR^–/–^, *n*_mice_ = 6. Data are presented as mean ± SEM. *P < 0.05 versus WT. Unpaired Student’s *t* test (2-tailed) was used for comparisons of 2 experimental groups in **C**–**F**, **H**, and **I**. Nonparametric, Mann-Whitney *U* test was used in **G**. Statistical comparison between dose-response curves was made by 2-way ANOVA with Bonferroni post hoc test in **J** and **K**.

To investigate the role of HuR in the development of CMD, we generated EC-specific *HuR*-KO mice (Tie2-HuR^–/–^) by crossing *HuR* exon-2 floxed mice (HuR^fl/fl^) with Tie2-driven Cre–overexpressing mice (Figure 3A). Figure 3B shows the typical genotyping result from the tail samples of WT, HuR^fl/fl^, Tie2-HuR^–/+^, and Tie2-HuR^–/–^ mice. Next, we examined *HuR* gene expression in CECs isolated from WT and Tie2-HuR^–/–^ mice. *HuR* deletion from ECs abolished *HuR* mRNA expression (Figure 3C) and HuR protein (Figure 3D) in mouse CECs. We further examined the HuR levels in other cell types and found that HuR deletion did not alter HuR levels in CMs (Figure 3E) and aortic SMCs (Figure 3F), but it significantly decreased HuR in monocytes (Figure 3G). There is no difference in body weight, blood glucose, and mean arterial pressure (MAP) between WT and Tie2-HuR^–/–^ mice (Table 2). However, Tie2-HuR^–/–^ mice displayed decreased CFVR (Figure 3H) and capillary density (Figure 3I), as well as increased apoptotic ECs compared with WT mice (Supplemental Figure 2). We found that there was no significant difference in apoptotic cell number in other cell types (total apoptotic cells – apoptotic ECs) between WT mice and Tie2-HuR^–/–^ mice (WT, 2.4 ± 0.4 NA/mm^2^; Tie2-HuR^–/–^, 2.9 ± 0.5 NA/mm^2^; *n*_mice_ = 5 per group), indicating that cell apoptosis induced by *HuR* deletion was confined to CECs. Furthermore, HuR deletion in ECs significantly attenuated EDR (Figure 3J) without altering endothelium-independent relaxation (Figure 3K). These data provide strong evidence that EC-specific deletion of HuR induces the reduction of capillary density and impaired EDR, ultimately attenuating coronary microvascular function.

### Target genes with altered expression in CECs from diabetic and Tie2-HuR^–/–^ mice.

**Figure 4:**
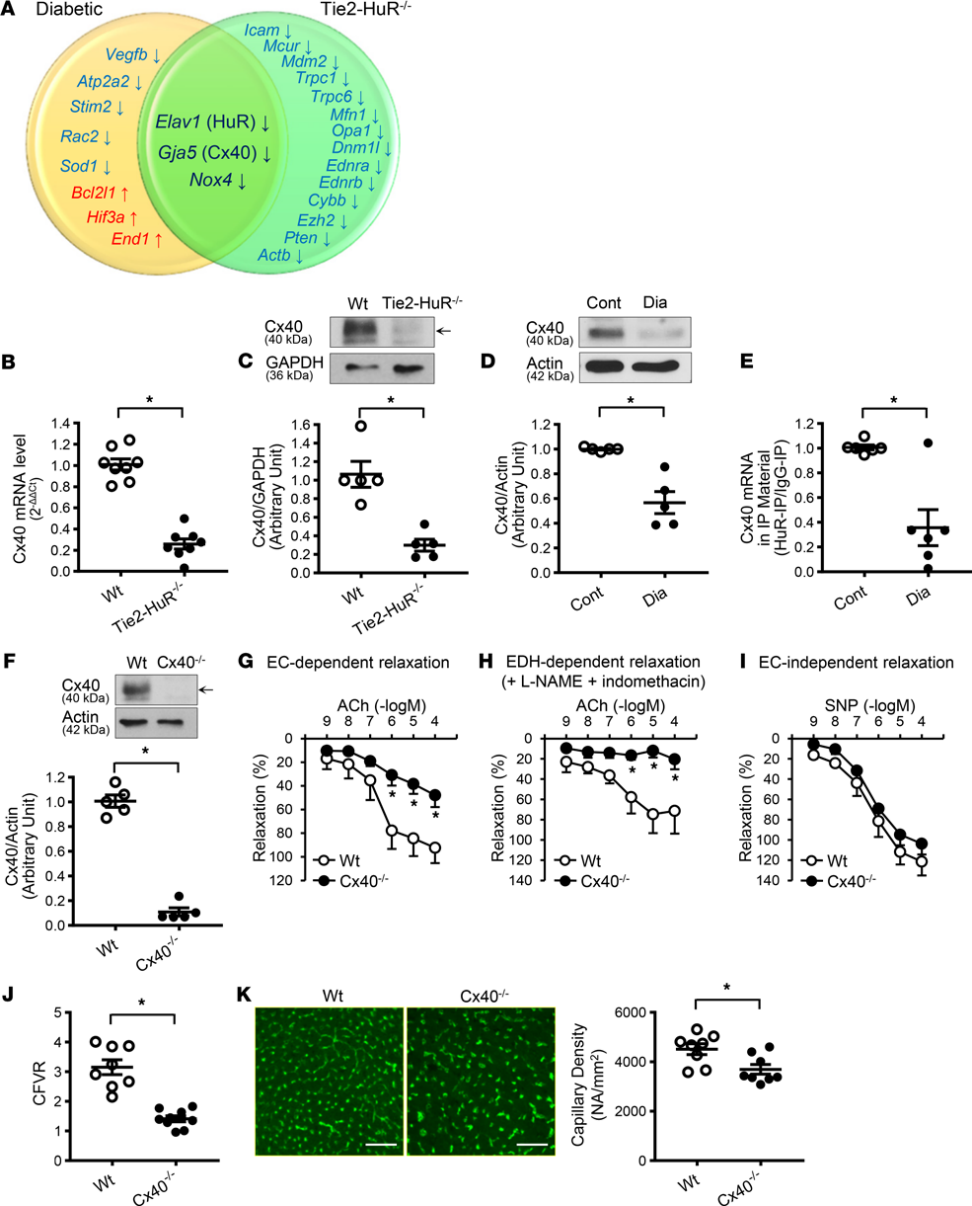
Identification of HuR-regulated genes and the effect of *Cx40* deletion on coronary microvascular function. (**A**) mRNA levels were compared between control and diabetic mice (*n*_experiments_ = 6 [12 mice per group]) and between WT and Tie2-HuR^–/–^ mice (*n*_experiments_ = 6 [12 mice per group]) (Supplemental Tables 4 and 5). Blue, downregulated genes; red, upregulated genes. (**B**) *Cx40* mRNA levels in CECs from WT and Tie2-HuR^–/–^ mice determined by real-time PCR. *n*_mice_ = 8 per group. (**C**) Western blot showing Cx40 and GAPDH protein levels in CECs from WT and Tie2-HuR^–/–^ mice (top panel). The bottom dot plot shows Cx40 protein level normalized to GAPDH. *n*_mice_ = 5 per group. (**D**) Western blots showing Cx40 and Actin protein levels in CECs from control and diabetic mice (top panel). The bottom dot plot shows Cx40 protein level normalized to Actin. *n*_mice_ = 5 per group. (**E**) Binding of *Cx40* mRNA to HuR protein determined by ribonucleoprotein immunoprecipitation. mRNA levels were determined by real-time PCR. *n*_mice_ = 6 per group. (**F**) Western blots showing Cx40 and Actin protein level in CECs from WT and Cx40^–/–^ mice (top panel). The bottom dot plot shows Cx40 protein level normalized to Actin. *n*_mice_ = 5 per group. (**G**) Endothelium-dependent relaxation evaluated by ACh-induced relaxation in CAs. *n*_mice_ = 7 per group. (**H**) Endothelium-dependent hyperpolarization–mediated (EDH-mediated) relaxation in CAs determined by ACh administration in the presence of L-NAME (an endothelial NO synthase inhibitor, 1 × 10^–4^M) and indomethacin (a cyclooxygenase inhibitor, 1 × 10^–5^M). *n*_mice_ = 7 per group. (**I**) Endothelium-independent relaxation evaluated by SNP-induced relaxation in CAs. *n*_mice_ = 7 per group. (**J**) CFVR. WT, *n*_mice_ = 8; Cx40^–/–^, *n*_mice_ = 9. (**K**) Representative photomicrographs (left) and summarized data (right) of capillary density. Scale bar: 50 μm. *n*_mice_ = 8 per group. Data are presented as mean ± SEM. *P < 0.05 versus Cont or WT. Statistical comparison between dose-response curves was made by 2-way ANOVA with Bonferroni post hoc test (**G**–**I**). Unpaired Student’s *t* test (2-tailed) was used for comparisons of 2 experimental groups (**B**–**F** and **J**–**K**).

To define the target genes altered by *HuR* deletion and by diabetes, we conducted the real-time PCR on 92 genes (Supplemental Table 3) and compared the mRNA levels of these genes between control and diabetic mice (Supplemental Table 4) and between WT and Tie2-HuR^–/–^ mice (Supplemental Table 5 and Figure 4B). The genes altered by diabetes and/or *HuR* deletion are summarized in a Venn diagram (Figure 4A). We chose the 92 genes that are expressed in ECs and play crucial roles in endothelial functions, such as (a) endothelium-derived relaxing factors and their regulators; (b) modifiers of cytosolic [Ca^2+^], mitochondrial [Ca^2+^], and endoplasmic reticulum [Ca^2+^]; and (c) regulators of EC proliferation, migration, and apoptosis. The expression levels of 3 genes — *Elav1* (*HuR*), *Gja5* (connexin40, *Cx40*), and nicotinamide adenine dinucleotide phosphate–reduced oxidases 4 (*Nox4*) — were significantly decreased in CECs from diabetic mice and in CECs from Tie2-HuR^–/–^ mice compared with their controls. These results demonstrate that a selective group of genes (*HuR*, *Cx40*, and *Nox4*) is concomitantly downregulated in mice with diabetes and mice genetically deleted endothelial HuR. The next set of experiments was designed to examine whether downregulated HuR in diabetic mice may directly regulate Cx40, an important gap junction protein required for normal EC function, to decrease coronary microvascular function.

### Downregulated Cx40 level and decreased Cx40 mRNA binding to HuR protein in CECs of diabetic mice.

We first confirmed that Cx40 protein level was downregulated in CECs from Tie2-HuR^–/–^ mice (Figure 4C) and diabetic mice (Figure 4D) compared with their controls. Next, we performed ribonucleoprotein immunoprecipitation (RIP) to examine the binding of *Cx40* mRNA to HuR protein (Supplemental Figure 6) and found that *Cx40* mRNA binding to HuR protein was significantly lower in CECs from diabetic mice than in CECs from control mice (Figure 4E). Taken together, downregulated Cx40 in diabetic EC is potentially due to reduced HuR level and decreased HuR binding to *Cx40* mRNA in diabetic mice.

### Deletion of Cx40 decreases CFVR by reducing capillary density.

Since HuR downregulation results in decreased Cx40 protein level, we examined the role of Cx40 in the development of CMD. Cx40 is a component of gap junction that acts as a tunnel for small molecules (<1 kDa) and electrical propagation during endothelium-dependent hyperpolarization–mediated (EDH-mediated) vascular relaxation. There was no difference in body weight between WT and Cx40^–/–^ mice; however, Cx40^–/–^ mice exhibited a slight increase in plasma glucose level and MAP (Table 2). Figure 4F demonstrated that Cx40 protein level was diminished in CECs of Cx40^–/–^ mice. EDR was assessed by ACh-induced relaxation, and EDH-mediated relaxation was evaluated by ACh-induced relaxation in the presence of L-NAME (an eNOS inhibitor) and indomethacin (a cyclooxygenase inhibitor). The concentration of PGF_2α_ used for precontraction was 5.34 ± 0.19 in WT and 5.63 ± 0.16 in Cx40^–/–^ mice (shown in –log[M]). The diameter of the vessels was 134.6 ± 7.1 μm in WT and 136.1 ± 5.4 μm in Cx40^–/–^ mice. There was no significant difference in either PGF_2α_ concentration or vessel diameter between WT and Cx40^–/–^ mice. We confirmed that EDR- (Figure 4G) and EDH-mediated relaxation (Figure 4H), but not EC-independent relaxation (Figure 4I), were significantly attenuated in CAs isolated from Cx40^–/–^ mice compared with WT mice. Those data indicate that deletion of *Cx40* sufficiently inhibited gap junction function. Importantly, Cx40^–/–^ mice exhibited a significant decrease in CFVR (Figure 4J) and capillary density (Figure 4K), along with an increase in EC apoptosis (Supplemental Figure 2) compared with WT. These results indicate that Cx40, which is directly regulated by HuR, is required for maintaining normal coronary endothelial function.

### Inhibition of HuR attenuates capillary network formation in control CECs, and overexpression of Cx40 increases capillary network formation in HuR-reduced CECs.

**Figure 5:**
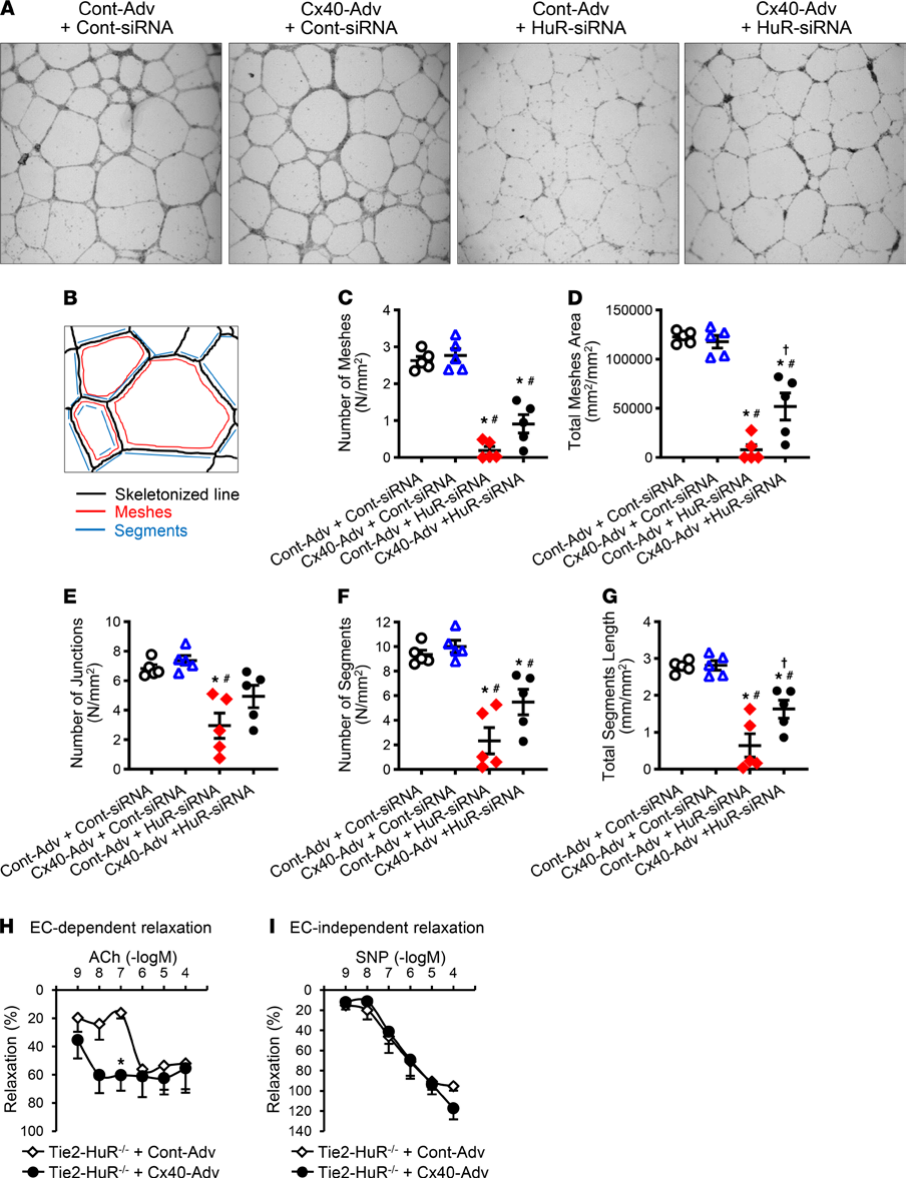
Overexpression of Cx40 improved endothelial function in HuR-inhibited CECs. (**A**–**G**) Effect of Cx40 overexpression and HuR inhibition on capillary network formation in human CECs. (**A**) Representative photograph image of capillary network. Original magnification, ×4. (**B**) Schematic diagram of capillary network parameters. (**C**–**G**) Summarized data of the number of meshes (**C**), total mesh area (**D**), junction numbers (**E**), number of segments (**F**), and total segments length (**G**) in human CECs with or without Cx40 overexpression (Cont-adenovirus [Cont-Adv] or Cx40-Adv, 100 pfu/cell, 96 hours) in the absence or presence of HuR inhibition (Cont-siRNA or HuR-siRNA, 100 nM, 72 hours). *n*_experiments_ = 5 per group. *P < 0.05 versus Cont-Adv + Cont-siRNA, *#P* < 0.05 versus Cx40-Adv + Cont-siRNA, †*P* < 0.05 versus Cont-Adv + HuR-siRNA. (**H** and **I**) Effect of Cx40 overexpression on vascular relaxation in Tie2-HuR^–/–^ mice. (**H**) Endothelium-dependent relaxation evaluated by ACh-induced relaxation in CAs. *n*_mice_ = 4 per group. (**I**) Endothelium-independent relaxation evaluated by SNP-induced relaxation in CAs. Tie2-HuR^–/–^ + Cont-Adv; *n*_mice_ = 4, Tie2-HuR^–/–^ + Cx40-Adv; *n*_mice_ = 3. *P < 0.05 versus Tie2-HuR^–/–^ + Cont-Adv. Data are presented as mean ± SEM. Statistical comparison between groups was made by 1-way ANOVA with Bonferroni post hoc test in **C** and **E**–**F**. Nonparametric, Kruskal-Wallis test, was used in **D**. Statistical comparison between dose-response curves was made by 2-way ANOVA with Bonferroni post hoc test (**H**, **I**).

To examine how *HuR* deletion and *Cx40* overexpression alter angiogenic capability of ECs, we conducted an ex vivo angiogenesis assay in human CECs. *Cx40* was overexpressed using Cx40-adenovirus (Cx40-Adv), and *HuR* was inhibited by HuR siRNA transfection in human CECs (Supplemental Figure 7). Figure 5, A–G, demonstrates that HuR inhibition in control CECs significantly reduced capillary network formation, suggesting that HuR regulates endothelial angiogenic capability. *Cx40* overexpression in control CEC did not affect angiogenic capability; however, *Cx40* overexpression in *HuR*-inhibited CECs slightly, but significantly, increased capillary network formation. Taken together, the decreased Cx40 due to HuR downregulation in CECs contributes to inhibiting EC-driven angiogenesis, reducing capillary density and ultimately attenuating coronary microvascular function.

### Cx40 gene transduction in CAs augments EDR in Tie2-HuR^–/–^ mice.

Cx40 overexpression was achieved by Cx40-Adv transduction in CAs dissected from Tie2-HuR^–/–^ mice. Twenty-four hours after transduction, CAs were mounted in the wire myograph, and EC-dependent and -independent relaxation was determined and compared between control-Adv and Cx40-Adv transduced CAs. As shown in Figure 5H, EDR was significantly increased by Cx40 overexpression, while EC-independent relaxation was not altered by Cx40 overexpression (Figure 5I).

### Overexpression of Cx40 in diabetic mice improves coronary microvascular function.

**Figure 6:**
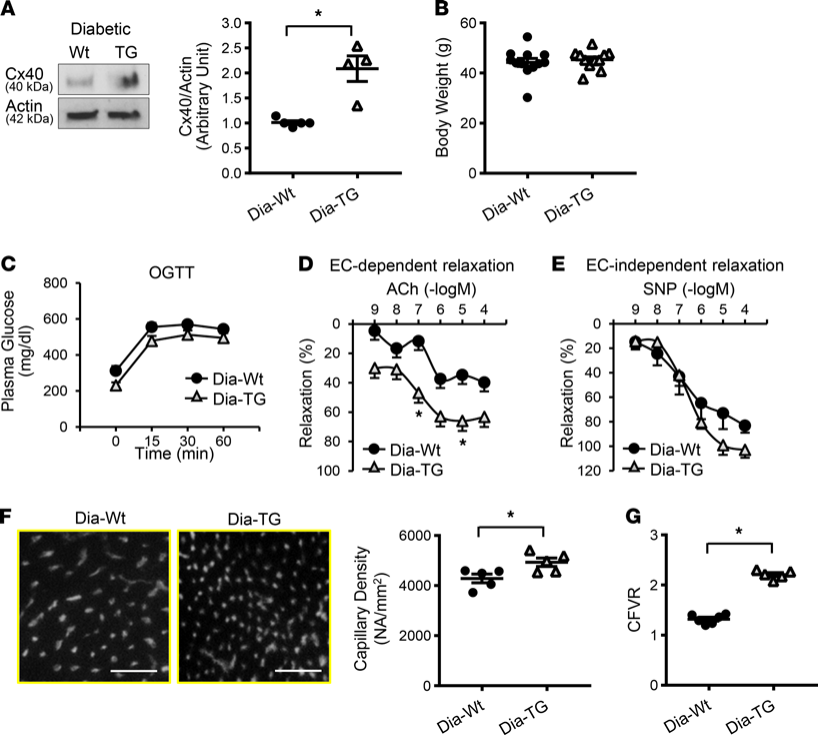
Cx40 overexpression in ECs increased CFVR and capillary density in diabetic mice. (**A**) Western blots showing Cx40 and Actin protein levels in CECs (left panel). The right dot plot shows Cx40 protein level normalized to Actin. Diabetic WT (Dia-WT), *n*_mice_ = 5; Cx40-overexpressing diabetic mice (Dia-TG), *n*_mice_ = 4. (**B**) Body weight. Dia-WT, *n*_mice_ = 12; Dia-TG, *n*_mice_ = 11. (**C**) OGTT. Dia-WT, *n*_mice_ = 12; Dia-TG, *n*_mice_ = 11. (**D**) Endothelium-dependent relaxation evaluated by ACh administration in CAs. Dia-WT, *n*_mice_ = 5; Dia-TG, *n*_mice_ = 7. (**E**) Endothelium-independent relaxation evaluated by SNP administration in CAs. Dia-WT, *n*_mice_ = 5; Dia-TG, *n*_mice_ = 7. (**F**) Representative photomicrographs (left) showing capillary density. ECs were stained by BS-lectin-TRITC since this strain also expresses EGFP. Scale bar: 50 μm. Averaged data (right) showing capillary density in Dia-WT (*n*_mice_ = 5) and Dia-TG (*n*_mice_ = 5) mice. (**G**) CFVR. Dia-WT, *n*_mice_ = 6; Dia-TG, *n*_mice_= 5. Data are presented as mean ± SEM. *P < 0.05 versus Dia-WT. Statistical comparison between time-dependent curves was made by 2-way ANOVA with Bonferroni post hoc test (**C**–**E**). Unpaired Student’s *t* test (2-tailed) was used for comparisons of 2 experimental groups (**A**, **B**, **F**, **G**).

T2D was induced in WT mice and EC-specific *Cx40*-overexpressing mice (43). The mice were used for the experiments 16 weeks after diabetic induction. Under diabetic conditions, *Cx40* transgenic mice demonstrated increased Cx40 protein levels in CECs compared with WT (Figure 6A). *Cx40* overexpression did not alter body weight (Figure 6B) or glucose tolerance (Figure 6C) in diabetic mice but decreased plasma glucose level at nonfasting conditions (Table 2). EDR (Figure 6D), but not smooth muscle–dependent relaxation (Figure 6E), was significantly augmented by Cx40 overexpression in CAs of diabetic mice. Furthermore, *Cx40* overexpression significantly increased capillary density (Figure 6F) and CFVR (Figure 6G) in diabetic mice. We here provide strong evidence that overexpression of Cx40 is sufficient to restore EC-dependent vasodilation, capillary density, and CFVR in diabetic mice. These observations suggest that increasing Cx40 expression and/or function is a potential strategy for treating CMD in diabetes in which HuR level is downregulated.

## Discussion

Clinical data indicate that patients with diabetes with CMD exhibit high cardiac mortality (19, 20); however, the molecular mechanisms by which diabetes leads to CMD are poorly understood. To investigate microvascular function in diabetes, we used an inducible T2D mouse model generated by administrating a single injection of low-dose STZ and with feeding a high-fat diet. This diabetic model has given us reproducible data with hyperglycemia and hyperinsulinemia (38, 40). T2D mice not only exhibited increased body weight and abnormal glucose tolerance, but also suffered from dyslipidemia (Figure 1, A and B, and Tables 1 and 2). However, lipid plaque formation has never been detected in this model. Therefore, the reduction of CFVR (Figure 1C) is due solely to coronary microvascular dysfunction, and decreased CFVR indicates that mice suffer from CMD. In line with the result from inducible T2D mice, TH mice (spontaneous T2D mice) exhibited reduced CFVR (18) without detectable plaque formation, suggesting that chronic hyperglycemia is the risk factor of CMD. Since capillary density positively correlates with coronary flow reserve (8, 44, 45), decreased capillary density in the heart could be a cause of reduced CFVR. Figure 1D demonstrates that T2D mice displayed decreased capillary density in the LV. Capillary density can be reduced by (a) augmented EC apoptosis, (b) attenuated cell migration and/or proliferation of neighboring mature ECs, and/or (c) reduced mobilization of circulating endothelial progenitor cells (EPCs) in sites of the vascular wall where ECs are damaged and/or lost. We and other investigators demonstrated that CECs are apoptotic in diabetes (12, 18, 46, 47) (Supplemental Figure 2) and are characterized by a diminished ability to migrate and proliferate in diabetes (18, 48). The number of EPCs is also decreased, and the function of EPCs is attenuated in patients with diabetes and diabetic animal models (49, 50). This study was designed to identify the key genes that influence capillary density and ultimately change microvascular function in the diabetic heart.

Like other RBPs, HuR binds to many RNAs and changes their fates. The data from systemic and tissue-specific deletion of *HuR* gene indicate that HuR plays a critical role in embryonic development and the regulation of physiological function (23–26). Therefore, it was natural to hypothesize that an abnormal level of HuR may be involved in the development or progression of cardiovascular disease. The conclusive data came from cancer research at first; HuR expression is increased with cancer and aids in the progression of angiogenesis in tumor tissue (31). However, the contribution of HuR to other diseases is still controversial. Zhou et al. demonstrated that HuR protein level was significantly reduced in the heart from the patients with heart failure compared with control patients (29). They also showed that myocardial infarction (MI) decreased HuR protein level in mouse hearts, and overexpression of *HuR* by AAV-*HuR* injection reduced infarct size and improved cardiac function (29). On the other hand, Krishnamurthy et al. found that HuR level was increased after MI in mice, and downregulation of HuR by *HuR*-shRNA lentivirus injection restored cardiac function (51). Unfortunately, we could not find the reason for the discrepancy between these studies. Recent reports demonstrate that HuR upregulation is implicated in the development of atherosclerosis (52, 53) and diabetic nephropathy (54). We found that HuR level was significantly decreased in CECs from diabetic mice compared with control mice (Figure 2, A and B). This phenomenon was also observed in CECs from a spontaneous T2D mouse model, TH mice (Figure 2C), and CECs from patients with diabetes (Figure 2D). Tie2-HuR^–/–^ mice exhibited similar microvascular functions to T2D mice (reduced CFVR, decreased capillary density, and increased EC apoptosis in the LV compared with WT; Figure 3 and Supplemental Figure 2), suggesting that decreased HuR expression in CECs is one of the leading causes of CMD in diabetes.

Although the Tie2-Cre mouse is commonly used to delete floxed genes in ECs, it has been reported that Tie2-Cre also exhibits an unneglectable level of recombination in the hematopoietic lineage (55). We found that *HuR* deletion using Tie2-Cre did not alter HuR expression levels in CMs and aortic SMCs (Figure 3, E and F) but significantly decreased the level in monocytes (a hematopoietic lineage cell) (Figure 3G). These data suggest that HuR-reduced monocytes might influence the functional change seen in Tie2-HuR^–/–^ mice. The potential role of HuR deletion in monocyte (or myeloid cell lineage) on endothelial and/or cardiac function can be identified using mice carrying HuR^fl/fl^ and LyzM-Cre in the future study.

Examining the effect of *HuR* overexpression on EC function in diabetic mice is believed to be essential. However, we encountered a problem when overexpressing *HuR* in CECs. In ex vivo studies, we found that the working concentration of *HuR* overexpression is very narrow. Overexpression of *HuR* easily killed CECs, and we had a very difficult time controlling HuR levels during the experiment. This implies that HuR may not only interact with mRNAs that are important for EC angiogenesis, but it may also bind to mRNAs that regulate cell death. Therefore, we decided to examine the target genes of HuR, which are also involved in CMD in diabetes. We examined 92 (Supplemental Table 3) genes by real-time PCR using a PCR plate custom-made by QIAGEN. We are aware that there are other genes that are not included in the plate but are also important for EC function. We did not use a microarray or RNA sequencing (RNAseq) in this study because (a) these experiments would require more animals to obtain a sufficient amount of mRNA, and (b) they would provide an overwhelming amount of information for the studies at the time. We believe that real-time PCR with 92 genes still gave us sufficient information to move to the next step. Interestingly, we found that only 3 genes out of 92 were altered in CECs by HuR deletion and diabetes: *HuR*, *Cx40*, and *Nox4* (Figure 4 and Supplemental Tables 4 and 5). Cx40 protein levels were significantly decreased in diabetes and Tie2-HuR^–/–^ mice compared with those controls, and HuR-bound *Cx40* mRNA was significantly lower in diabetes than in control (Figure 4, B–D). These data indicate that Cx40 could be a potential target of HuR. It is important to note that we examined the protein level of Nox4 and found that Nox4 protein level was not altered in CECs of diabetic mice compared with the control (control, 1.01 ± 0.06; diabetic, 1.16 ± 0.32. *n*_mice_=7 per group. *P* = 0.65). Therefore, we did not conduct a further experiment to examine the role of Nox4 in this study. We were indeed surprised to see that only 3 genes were shared in diabetic and Tie2-HuR^–/–^ mice after screening genes in an unbiased way. These results suggest that HuR overexpression in diabetic mice may not be ideal, since it would potentially lead to unnecessary alterations in many genes besides Cx40. Thus, we believe overexpression of Cx40, a downstream HuR-sensitive gene, in diabetes could be a safer and better option to treat diabetic cardiovascular complications than modification of HuR.

Cx40 is a major gap junction protein in ECs, and decreased gap junction activity due to reduced Cx40 expression attenuates EDH (56) and endothelial migration (57). We and other investigators demonstrated that ECs in type 1 diabetic mice exhibited a significant decrease in Cx40 protein level (11, 58). However, there is no report examining the role of Cx40 in CECs of T2D mice, to the best of our knowledge. The results from Figure 4 suggest that HuR regulates *Cx40* gene expression in CECs, and decreased HuR protein level and HuR binding to *Cx40* mRNA are, at least in part, the causes for downregulated Cx40 expression in CECs in diabetes. Therefore, we obtained *Cx40^–/–^* mice (59) (Figure 4F) to examine whether mice without Cx40 exhibit similar coronary microvascular function shown in diabetes. First, we examined EDH-mediated relaxation in the third order of CAs to show the functional change of gap junction by Cx40 deletion. EDH-mediated relaxation was significantly attenuated by *Cx40* deletion in CAs (Figure 4H) without any change in smooth muscle–dependent relaxation (Figure 4I), suggesting that *Cx40* deletion is functionally working. Note that EDH-mediated relaxation was evaluated in this study, but endothelial-derived hyperpolarization factor–induced (EDHF-induced) relaxation was not. Certain EDHFs can evoke SMC relaxation by direct activation of K^+^ channels in SMCs. In other words, EDHFs could relax vessels without EC hyperpolarization and its electrical propagation through gap junctions. Cx40 deletion significantly attenuated ACh-dependent relaxation in the presence of L-NAME; however, the vessels still relaxed by about 20% (Figure 4H and Supplemental Figure 8), implying that the rest of relaxation would be induced by EDHFs via direct hyperpolarization of SMCs. Next, we examined coronary microvascular function and found that CFVR was significantly reduced in *Cx40^–/–^* mice (Figure 4J), accompanied by decreased capillary density and increased EC apoptosis (Figure 4K and Supplemental Figure 2). This is the first report to our knowledge to demonstrate that the loss of Cx40 leads to CMD. We, therefore, hypothesized that the CMD seen in T2D mice might result from decreased Cx40 expression in CECs due to downregulated HuR expression.

MAP in *Cx40^–/–^* mice was slightly, but significantly, higher than in WT mice (116.6 mmHg versus 99.0 mmHg). To test whether reduced CFVR in *Cx40^–/–^* mice is due to Cx40 gene deletion or caused by this slight increase in MAP, we examined CFVR in Tie2-driven *Cx40* negative mutant overexpressing (Cx40^NM^) mice (43). As shown in Supplemental Figure 9, MAP in Cx40^NM^ mice was similar to the level in WT mice; however, CFVR was significantly decreased in Cx40^NM^ mice, implying that Cx40 deletion, but not MAP increase, leads to the reduction of CFVR in *Cx40^–/–^* mice.

It has been known for decades that coronary endothelial dysfunction is implicated in the development of obstructive CAD. However, the Women’s Ischemia Syndrome Evaluation (WISE) study shed light on endothelial dysfunction in patients with nonobstructive CAD (CMD) in 2004. Their data suggest that the decrease in coronary microvascular function predicts adverse cardiovascular outcomes independently of CAD severity (60). In addition, the treatment with ranolazine (a late sodium current inhibitor that is commonly used for obstructive CAD) does not show any beneficial effect on ischemia in patients with CMD (61). These reports emphasize the necessity to develop new drugs specific for patients with CMD. We believe that Cx40 is an excellent therapeutic target for CMD based on the following reasons: (a) there are more myoendothelial gap junctions that are composed of connexins (Cxs) in smaller resistant vessels than in large vessels (62–64), suggesting that Cx40 upregulation could be more effective in small vessels; (b) Cx40 is predominantly expressed in ECs (62, 65), so increased Cx40 expression and activity lead to a specific effect on EC function; and (c) overexpression of Cx40 does not lead to abnormal angiogenesis as shown in tumor tissues (66). We demonstrate here that overexpression of Cx40 in ECs augments EDR, increases capillary density, and results in improved CFVR in diabetic mice (Figure 6). These results provide strong evidence that overexpression of Cx40 is a useful therapeutic strategy for CMD in diabetes.

Overexpression of Cx40-augmented angiogenesis in HuR-deficient CECs ex vivo (Figure 5) and increased capillary density in vivo (Figure 6F). Other investigators also show that Cx40 positively regulates cell migration and angiogenesis (57); however, the detailed mechanisms of how exactly Cx40 enhances angiogenesis are still unknown. It has been reported that Cx43 overexpression increases (67, 68) or decreases (69, 70) angiogenesis or cell migration independently of gap junction activity. The deletion of Cx37 promotes angiogenesis (71). These data suggest that the effect of Cxs on cell migration and angiogenesis seems to be different among the subtypes of Cxs and that enhanced EC angiogenesis by *Cx40* overexpression may be due not only to increased gap junction activity, but also to unknown mechanisms through *Cx40* overexpression. It has been reported that Cx43 regulates the expression of other genes (72); therefore, it is possible that Cx40 can also regulate the expression of other genes.

We found that diabetic mice, Tie2-HuR^–/–^ mice, and *Cx40^–/–^* mice exhibited reduced EDR in CAs compared with their controls (Figure 1E, Figure 3J, and Figure 4G), and overexpression of Cx40 in diabetic mice augmented EDR (Figure 6D). Attenuated relaxation in small coronary vessels contributes to coronary microvascular dysfunction and leads to CMD. Our vessels were obtained from the third and fourth order of CAs, which might not be classified as “small vessels,” but these are the smallest CAs from mice that we could mount in a myograph. We believe attenuated EDR in these CAs is an important contributor to the development of CMD in diabetes.

*HuR* or *Cx40* deletion in ECs led to endothelial apoptosis (Supplemental Figure 2). In this study, we focused on angiogenic capability of ECs (Figure 5) rather than endothelial apoptosis. However, increased cell apoptosis by *HuR* or *Cx40* deletion would contribute to decreased capillary density in the heart. Excess production of ROS is one of the leading causes of cell apoptosis. We previously reported that ROS formation in CECs was considerably increased in diabetes (12, 40, 73, 74). Supplemental Figure 10 demonstrates that the deletion of *HuR* or *Cx40* gene increased cytosolic ROS formation in human CECs. Excess ROS production by HuR inhibition was somewhat expected, since HuR deletion significantly downregulates Opa1 expression (Figure 4 and Supplemental Table 5). Opa1 is a mitochondrial fusion protein, and reduced mitochondrial fusion (or increased mitochondrial fission) increases mitochondrial ROS formation, followed by the rise of cytosolic ROS concentration (75). Increased ROS generation by Cx40 inhibition surprised us and would require further experiments to identify the molecular mechanisms. Other investigators have also investigated the potential mechanisms in which the inhibition of HuR leads to cell apoptosis. HuR binds to Mdm2, a primary negative regulator of p53; therefore, deletion of HuR increases p53 and leads to cell apoptosis (76). Inhibition of HuR also increases caspase 3 expression (26) and other proapoptotic factors (76) that ultimately induces cell apoptosis. We have reported that p53 is one of the major causes of coronary endothelial apoptosis in diabetes, and inhibition of p53 improves coronary microvascular function (18). It has to be noted that the mechanisms to induced EC apoptosis in Tie2-HuR^–/–^ and diabetic mice might be the same or different; therefore, it requires additional experiments to identify detailed molecular mechanisms in which *HuR* deletion– and hyperglycemia-induced endothelial-apoptosis in the heart.

We found that Cx40^–/–^ mice exhibited a significant increase in plasma glucose level, and *Cx40* overexpression in diabetes displayed a slight but significant decrease in plasma glucose level (Table 2). In the endocrine system, Cx40 is well known to regulate the function of renin-producing cells in the kidneys. Therefore, the increase in blood pressure by *Cx40* deletion (Table 2) might be partly led by increased renin secretion (77). However, there is no report showing that Cx40 contributes to insulin secretion and/or glucose tolerance. Cx36 is expressed in β cells, and *Cx36*-KO mice develop glucose intolerance via attenuation of glucose-stimulated insulin secretion from the β cells (78). Although Cx40 is not expressed in β cells, there may be mechanisms by which Cx40 regulates plasma glucose level (e.g., altered infiltration of inflammatory cells in adipose tissues). We will further investigate this phenomenon in future studies.

This study demonstrates for the first time to our knowledge that diabetes leads to downregulation of HuR, an RBP, which subsequently decreases expression of Cx40, a gap junction channel protein, in cardiac ECs and attenuates coronary microvascular function. Overexpression of Cx40 increases capillary density and restores coronary microvascular function determined by CFVR in diabetes. These data indicate that Cx40 is a promising therapeutic target for developing novel and unique treatment for CMD in patients with diabetes.

## Methods

A list of materials used is available in the supplemental material.

### Animals.

C57BL/6NHsd male mice were purchased from Envigo RMS Inc. Inducible T2D mice were generated by a single injection of STZ (75 mg/kg, dissolved in citrate buffer, i.p.) at 6 weeks of age and given a high-fat diet (60% kcal from fat, Envigo RMS Inc.) from the day of STZ injection. Sixteen weeks after diabetic induction, mice were randomly allocated to experimental groups and used before 26 weeks old (20 weeks after T2D induction). Oral glucose-tolerance test (OGTT) and measurements of plasma cholesterol, HDL, and triglyceride levels were performed as described previously (18, 38, 40). OGTT was conducted 6 hours after fasting. Male TH mice were purchased from The Jackson Laboratory and bred in our animal facility. The TH mice are a polygenic T2D model, and male TH mice exhibit hyperglycemia, hyperinsulinemia, hyperlipidemia, and obesity (18). We used male C57BL/6 mice as WT controls according to The Jackson Laboratory guidelines. All mice were fed with a normal laboratory diet (13% kcal from fat, Lab Diet). TH and WT mice were used for experiments at the age of 16–20 weeks.

HuR^fl/fl^ mice were provided by Jian-Ying Wang from the University of Maryland at Baltimore (Baltimore, Maryland, USA) (25) and crossed with Tie2-Cre mice (The Jackson Laboratory) to generate EC-specific *HuR*-KO mice (Tie2-HuR^–/–^ mice; Figure 3A). HuR^WT/WT^ mice were used as WT control for Tie2-HuR^–/–^ mice. Hetero (Tie2-HuR^+/–^) parents were used for the breeding due to the infertility of homo (Tie2-HuR^–/–^) parents. Genotype rates were: homozygous (17.4%), heterozygous (29.9%), and WT (52.7%) (Supplemental Figure 11). WT and Tie2-HuR^–/–^ mice were used after 16 weeks old. Systemic *Cx40*-KO (Cx40^–/–^) mice were provided by Janis Burt from the UA (59), and C57BL/6J (background strain, The Jackson Laboratory) were used as a WT control. They were used at 16 weeks old. Tie2-driven WT *Cx40*-overexpressing (Cx40^Tg^) mice and Tie2-driven *Cx40*^–^ mutant–overexpressing (Cx40^NM^) mice were provided by Anthony Ashton from the University of Sydney (43). These mouse strains carry the Tie2-driven WT Cx40 gene or Cx40^NM^ gene with EGFP (Cx40-IRES-EGFP); therefore, the mice will constitutively express WT Cx40 gene or Cx40^NM^ gene in ECs. Heterozygous parents were identified by copy number measurement and used as a breeder of Cx40^Tg^ mice, and homozygous parents were used for Cx40^NM^ breeders. Mice without *Tie2-Cx40* gene were used as a WT control of Cx40^Tg^ and Cx40^NM^ mice. These mice were bred in the animal facility of the UA and UCSD. Male Cx40^Tg^ mice were used for experiments between 22 and 26 weeks old (16–20 weeks after diabetic induction). The primer sequence information for genotyping is listed in Supplemental Table 1 and, for real-time PCR, in Supplemental Table 2. Heart dissection was performed under anesthesia with a mixture of ketamine (100 mg/kg, i.p.) and xylazine (5 mg/kg, i.p.), and all efforts were made to minimize pain.

The age was matched between diabetic or transgenic mice and their control mice. Male mice were used in this study due to the difference in the onset of hyperglycemia and diabetic complications between male and female mice.

### CFVR measurement.

CFVR was used to assess coronary microvascular function (18), instead of coronary flow reserve, because of the difficulty in precisely measuring coronary arterial diameter in mice (41). Coronary flow velocity (CFV) was measured using a Vevo 2100 system (FUJIFILM Visual Sonics Inc.; Supplemental Figure 1). Mice were anesthetized with isoflurane and kept on the heating pad at 37°C. The resting level of CFV was obtained at 1% isoflurane. CFVR was defined as maximal hyperemic CFV (induced by 2.5% isoflurane) divided by resting CFV (1% isoflurane) (18, 42). Each experiment was completed within 40 minutes, and the heart rate was kept above 400 bpm. If the procedure took longer, or the heart rate was dropped lower than the criteria, the data were eliminated without analysis.

### Immunofluorescence experiment.

The capillary density and EC apoptosis were evaluated in the LV as described previously (11, 12, 18). Briefly, the heart was dissected, embedded in OCT compound, frozen in 2-methylbutane precooled with liquid nitrogen, and then kept at –80°C until being sectioned with CryoStar NX70 Cryostat (Thermo Fisher Scientific). Sections (6 μm in thickness) were fixed in 4% formaldehyde for 5 minutes, blocked with 5% BSA for 30 minutes, and incubated with Bandeiraea Simplicifolia lectin-FITC (BS-l, Sigma Aldrich) for 30 minutes. BS-l was used to probe the terminal β-galactosyl saccharides associated with ECs on the surface of arterioles and venules, as well as capillaries. Apoptotic cells were detected using a TUNEL assay (an in situ cell death detection kit, Roche). For HuR imaging in CECs, cells were stained with HuR antibody and followed by anti–mouse Alexa488 (Supplemental Figure 12). The images were captured with a Nikon Eclipse Ti-E 3D Deconvolution microscope (Nikon Corp.) with a ×20 objective lens (for EC apoptosis and capillary density) or ×60 objective lens (for HuR staining) in a blinded fashion. The fluorescence intensities were calculated using ImagePro-PLUS 7.0 software (Media Cybernetics Inc.).

### Isometric tension measurement in coronary arterial ring.

Isometric tension measurement in CAs was performed as described previously (11, 38, 40). Briefly, third- or fourth-order small CAs were dissected from the hearts and then cut into 1 mm segments (Supplemental Figure 3). The CA rings were mounted on a myograph (DMT-USA Inc.) using thin stainless wires (20 μm in diameter), and the resting tension was set at 100 mg. CAs were allowed to equilibrate for 45 minutes with intermittent washes every 15 minutes. After equilibration, each CA ring was contracted by treatment with PGF_2α_ to generate a similar contraction level in all groups. ACh or sodium nitroprusside (SNP, an NO donor) was administrated in a dose-dependent manner (1 nmol/L to 100 μmol/L) (Supplemental Figure 4). The degree of relaxation was shown as a percentage of PGF2α-induced contraction.

### Isolation of mouse CECs.

Mouse CECs were isolated using a method previously described (11, 12, 18, 40). Briefly, after flushing blood from the heart, the heart was dissected, minced, and incubated with M199 containing 1 mg/mL collagenase II and 0.6 U/mL dispase II for 1 hour at 37°C. The digested material was collected and incubated with magnetic beads that were prepared as follows: Dynabeads sheep anti–rat IgG were incubated with rat anti–mouse CD31 monoclonal antibody (1 μg/mL) at 4°C overnight. The cell suspension was incubated with beads for 1 hour at 4°C, and then CECs were captured and isolated by the Dynal magnet (Thermo Fisher Scientific). The purity of the CEC population in cells isolated from hearts was tested by DiI-acLDL (Thermo Fisher Scientific) uptake and BS-l or CD144 staining (Supplemental Figure 13). Efficient isolation yields approximately 1 × 10^4^ cells from 1 heart, with over 80% purity. Western blot and real-time PCR were conducted with freshly isolated CECs from mice. For the IHC experiment, we cultured CECs after isolation, and experiments were performed within 5 days without passing the cells.

### Human CECs.

Human CECs from 4 control and 4 T2D patients were purchased from commercial suppliers (Supplemental Materials) and cultured in EC media composed of M199 supplemented with 10% FBS, 100 U/mL penicillin, 100 μg/mL streptomycin, 20 μg/mL ECGS, and 16 U/mL heparin. All experiments were conducted before passage 10.

### Isolation of mouse CMs and aortic smooth muscle cells.

Mouse CMs were collected after removing ECs from the digested materials of the hearts. After removing ECs, the majority of cells in the digested material are CMs; however, other types of cells (i.e., smooth muscle cells [SMCs] and fibroblasts) might be present in the samples at a very small percentage. The samples of aortic SMCs were obtained from an aorta after removing ECs by gently scrubbing the inner layer of the aortic lumen using a cotton tip.

### Western blot analysis.

Protein levels were analyzed using SDS-PAGE separation and electrophoretic transfer to nitrocellulose membranes. Primary antibodies used in this study are listed in Supplemental Materials.

### Real-time PCR.

mRNA from mouse CECs was isolated using a miRNeasy Mini Kit (QIAGEN), and cDNA was made by RT^2^ First Strand Kit (QIAGEN). We chose 92 genes (including *Actb* and *Gapdh*) that are highly expressed in ECs and play crucial roles in endothelial functions for analysis by real-time PCR, including (a) endothelium-derived relaxing factors and their regulators; (b) modifiers of cytosolic Ca^2+^ concentration ([Ca^2+^]), mitochondrial [Ca^2+^], and endoplasmic reticulum [Ca^2+^]; and (c) regulators of EC proliferation/migration/apoptosis (see Supplemental Table 3 for the gene list). The custom PCR plates were made by QIAGEN based on the selected genes (SABIO no. CAPA38128-6:CLAM25240). One 384-well plate includes quadruplicate wells for 1 gene (for the gene of interest and internal control) and replicates genomic DNA controls, reverse-transcription controls, and positive PCR controls. Primer sets used for the PCR plate are authenticated by the company. Real-time PCR was conducted using the CFX384 Touch Real-Time PCR Detection System (Bio-Rad Laboratories). GAPDH was used as an internal control. The transcript levels of the gene of interest were quantified according to the ΔCt method. Ct values > 35 were not included in the analysis and were considered as negative. Note that the primer set for *Elavl1* (HuR) on the plate (product no. PPM30921A, QIAGEN) detects exon 5, not exon 2; therefore, real-time PCR was repeated using an exon 2-specific primer (Supplemental Table 2).

### RIP.

To assess the association of endogenous HuR protein with endogenous *Cx40* mRNA, IP of ribonucleoprotein complexes was performed as previously described (25) (Supplemental Figure 4). Mouse CECs were isolated and lysed with lysate buffer (100 mM KCl, 5 mM MgCl_2_, 10 mM HEPES [pH 7.0], 0.5% Igepal, 1 mM DTT, 1% protease inhibitor cocktail, 1% phosphatase inhibitor cocktail, 100 U/mL in RNase free water). Prior to RIP, the IP matrix was conjugated with HuR antibody or IgG, and cell lysate was incubated with IP matrix overnight. RNA in IP materials was used for reverse transcription, followed by real-time PCR analysis (Figure 4). The data of *Cx40* mRNA bound to HuR protein was normalized by *Cx40* mRNA bound to IgG.

### Ex vivo angiogenesis assay in human CECs.

We used Cx40-Adv to overexpress the *Cx40* gene (11). HuR downregulation was achieved by HuR siRNA transfection (Santa Cruz Biotechnology Inc.). Human control CECs (1 × 10^5^ cells) were seeded on a 3 cm plate, and control-Adv or Cx40-Adv was added to the cells at the titer of 100 pfu/cell the following day. Twenty-four hours later, the viruses were washed, and cells were transfected with control siRNA or HuR siRNA at 100 nM using lipofectamine 3000 reagent (Thermo Fisher Scientific). Specific protein knockdown was verified with Western blotting 48 hours after transfection (Supplemental Figure 5). For ex vivo angiogenesis assay (18), cells were detached, and 4 × 10^4^ cells were seeded on the Matrigel-coated 4-well chamber. Twenty-four hours after plating cells, 4 microscopic fields, selected at random, were photographed using an EVOS FL Auto Cell Imaging System with a 4× objective lens (Thermo Fisher Scientific) in a blinded fashion. Mesh number, total mesh area, junction number, segments number, and total segments length were analyzed using Angiogenesis Analyzer in NIH ImageJ 1.51k software.

### Cytosolic ROS measurement in human CECs.

HuR and Cx40 were downregulated using HuR siRNA or Cx40 siRNA, respectively (Santa Cruz Biotechnology Inc.). Human control CECs (2 × 10^4^ cells) were seeded on a 4-well glass chamber and then transfected with control, HuR, or Cx40 siRNA at 100 nM using lipofectamine 3000 reagent. Cytosolic ROS was detected using the fluorescent probe dihydroethidium (DHE). Cells were preloaded with 50 μmol/L DHE for 30 minutes before capturing images. Cytosolic DHE exhibits blue fluorescence; once it is oxidized by ROS, it illuminates red (ethidium bromide [EB]). The images were captured with a Nikon Eclipse Ti-E 3D Deconvolution microscope with a 60× objective lens in a blinded fashion. The fluorescence intensity was calculated using ImagePro-PLUS 7.0 software. The background fluorescence intensity was subtracted from the cell intensity. The index of cytosolic ROS concentration is described as a ratio of EB and DHE.

### Statistics.

We conducted data analysis in a blinded fashion wherever possible and set proper controls for every experimental plan. The mouse numbers and independent experiment numbers are described in the figure legends. Statistical analysis was performed using GraphPad Prism 9. Data are presented as mean ± SEM. After the data passed a normality test (Shapiro-Wilk or Kolmogorov-Smirnov test), the 2-tailed Student’s *t* test was used for comparisons of 2 groups, and 1-way ANOVA was used for multiple comparisons. If the data did not pass the normality test, a nonparametric test (Mann-Whitney for 2 groups, Kruskal-Wallis for multiple comparisons) was used. Bonferroni’s multiple comparisons test was used as a post hoc test for one-way ANOVA and Dunn’s test for the Kruskal-Wallis test. Statistical comparison between dose-response curves was made by 2-way ANOVA with Bonferroni post hoc test. Differences were considered to be statistically significant when *P* < 0.05.

### Study approval.

All experimental protocols used in this study were approved by the IACUC at The UA and the UCSD and conformed to the *Guide for the Care and Use of Laboratory Animals* (National Academies Press, 2011). The universities have been certified by Public Health Service with Animal Welfare Assurance number A3248-01 (UA) and A3033-01 (UCSD); the approved IACUC protocol numbers for this study are 14-520 (at UA) and S18185 (at UCSD). The laboratory personnel who conducted experiments took all training required for animal handling and were certified by the IACUC.

## Author contributions

RS and JTOC conducted the experiments, analyzed the data, and drafted and reviewed the manuscript. ATH, RG, MW, LG, YSL, JSM, and JNR conducted the experiments and reviewed the manuscript. BTS, JW, AWA, JYW, and JXJY reviewed and edited the manuscript. AM conceived the project, designed the experiments, analyzed the data, and reviewed and edited the manuscript.

## Supplementary Material

Supplemental data

## Figures and Tables

**Table 1:**
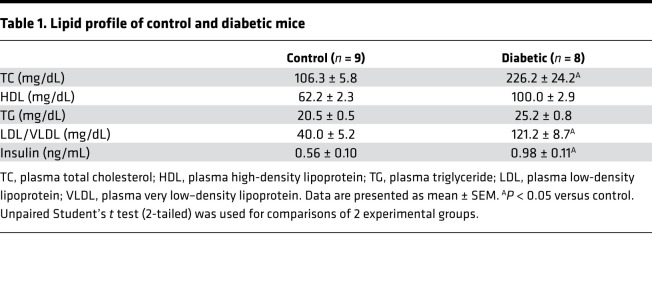
Lipid profile of control and diabetic mice

**Table 2:**
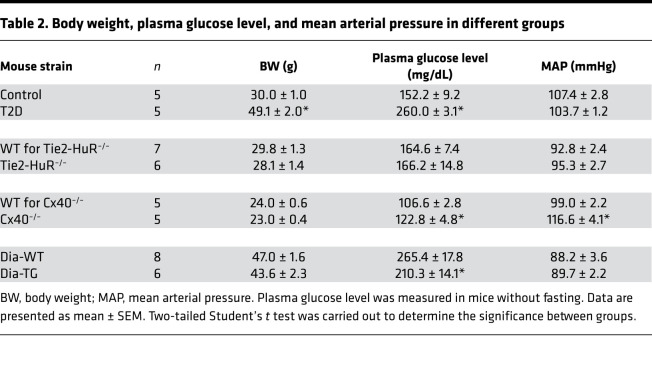
Body weight, plasma glucose level, and mean arterial pressure in different groups

## Version 1 11/08/2021

Electronic publication
