# Supplementary material for: HuR/Cx40 downregulation causes coronary microvascular dysfunction in type 2 diabetes
Source: JCI Insight. 2021 Nov 8;6(21):e147982. doi: 10.1172/jci.insight.147982 (PMC8663561; doi:10.1172/jci.insight.147982)
Supplement: Supplemental data [file jciinsight-6-147982-s161.pdf]

Supplemental Figures

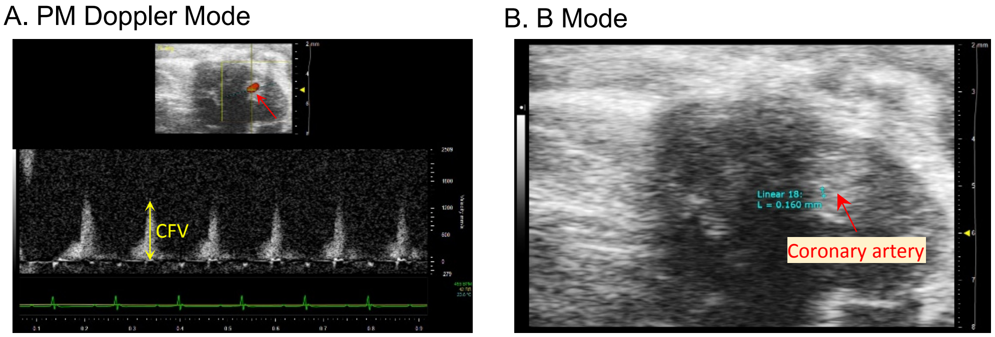

Figure S1. Representative image of coronary flow measurement. **A**: PM Doppler Mode. **B**: B-Mode. Coronary flow velocity reserve (CFVR) was used to assess coronary microvascular function. Coronary blood flow velocity (CFV) was measured using a Vevo 2100 system. The resting level of CFV was obtained at 1% isoflurane. CFVR was defined as maximal hyperemic CFV (induced by 2.5% isoflurane) divided by resting CFV (1% isoflurane).

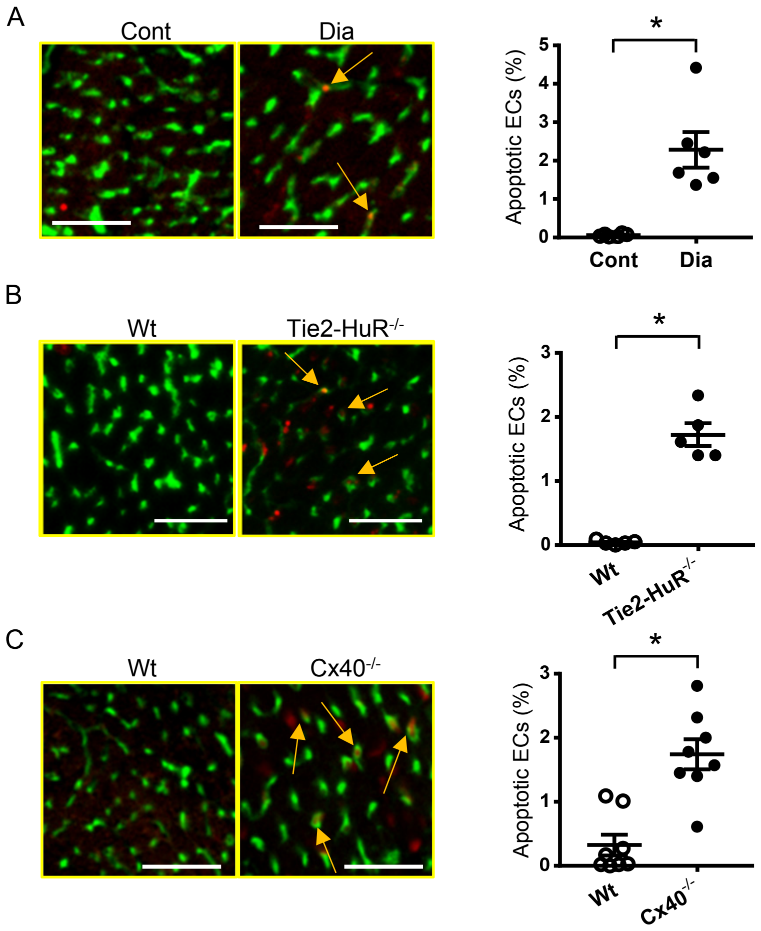

Figure S2. Increased endothelial apoptosis in the left ventricle of diabetic mice, Tie2-HuR-/- mice, and Cx40 KO mice compared to their controls. **A**: Representative photomicrographs (left) showing apoptotic ECs in the left ventricle of control and diabetic mice. ECs were stained by BS-lectin-FITC (green) and apoptotic cells were detected by TUNEL staining (red). An arrow indicates co-stained cells (apoptotic ECs, orange). Bar=50μm. Averaged data (right) showing the percentage of apoptotic ECs (the number of apoptotic ECs divided by total number of ECs). N_{mice}=6 for each group. **B**: Apoptotic ECs in Wt and Tie2-HuR$^{-/-}$ mice. N_{mice}=5 per group. **C**: Apoptotic ECs in Wt and Cx40$^{-/-}$ mice. N_{mice}=8 per group. Data are mean ± SEM. *P<0.05 vs. Cont or Wt. Unpaired Student's *t*-test (2-tailed) was used for comparisons of two experimental groups.

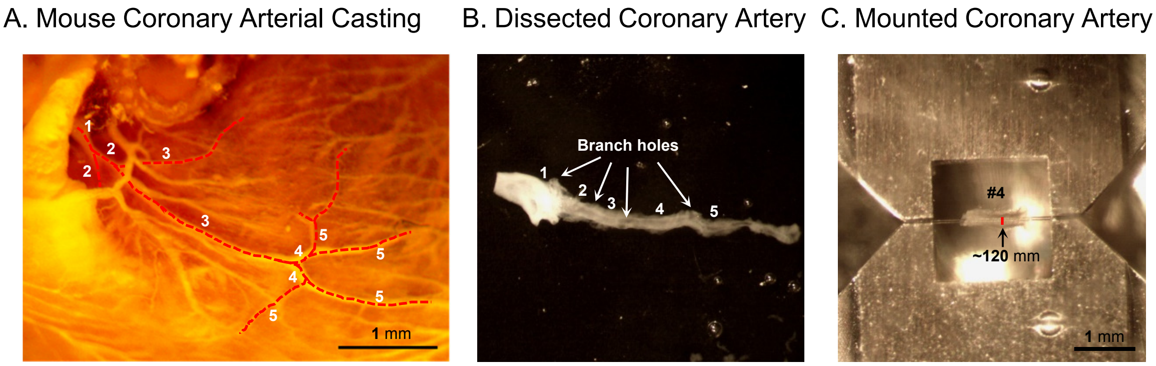

Figure S3. Mouse coronary arterial images. **A**: Mouse coronary arterial casting with microfil. The lines with red dots indicate the left anterior descending artery. The number in the picture indicates the order of branches. **B**: Dissected mouse coronary artery (CA). The arrows indicate holes after removing branches. **C**. Mounted mouse CA (branch #4 from image B) with 20 μm stainless wires in the DMT myograph.

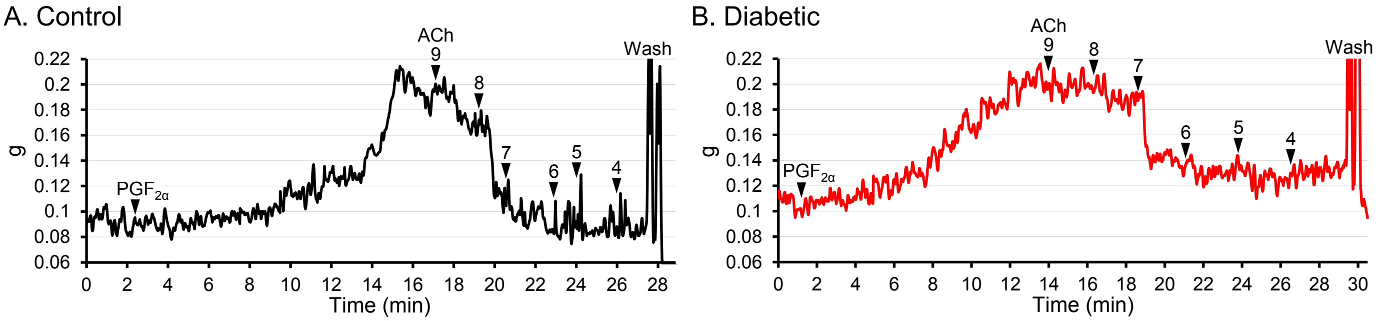

Figure S4. Representative record of endothelium-dependent relaxation (EDR) in CAs. $PGF_{2\alpha}$ was applied to generate ~0.1 g contraction. After precontraction, acetylcholine (ACh) was applied in a dose dependent manner from 10^{-9} M (9) to 10^{-4} M (4). After observed maximum relaxation, vessels were washed**. A**: CA dissected from control mouse. **B**: CA from diabetic mouse.

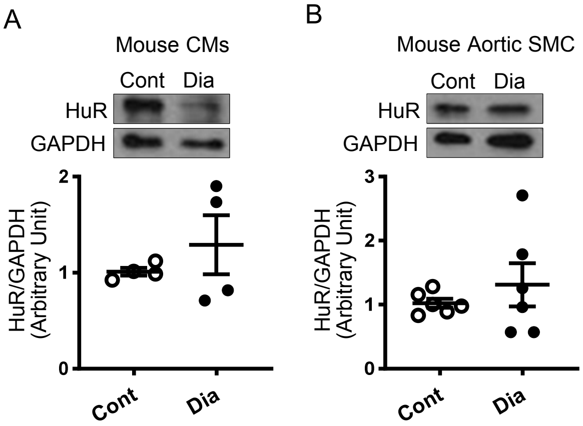

Figure S5. HuR protein level in control and diabetic mice determined by Western blots. **A**: Cardiac myocytes (CM, digested heart materials after depletion of ECs), n_{mice}=4 per group. **B**: aortic smooth muscle cells (SMCs), n_{mice}=6 per group. Data are mean ± SEM. Unpaired Student's *t*-test (2-tailed) was used for comparisons of two experimental groups.

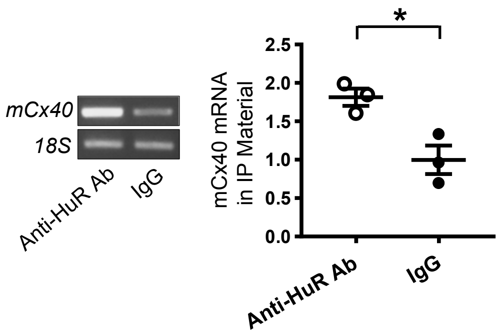

Figure S6. Ribonucleoprotein immunoprecipitation (RIP) to determine the level of Cx40 mRNA bound to HuR protein in mouse CECs. Association of endogenous HuR with endogenous Cx40 mRNA was measured by RIP/RT-PCR analysis using either anti-HuR antibody (Ab) or control IgG conjugated with IP matrix. Summarized data shows Cx40 mRNA levels divided by 18s level (input RNA without RIP). The signal of Cx40 mRNA bound to HuR protein is significantly higher than the level of Cx40 mRNA bound to IgG, indicating that HuR protein binds to Cx40 mRNA. $N=3_{experiment}$ per group. Data are mean ± SE. **P*<0.05 vs. IgG. Unpaired Student's *t*-test (2-tailed) was used for comparisons of two experimental groups.

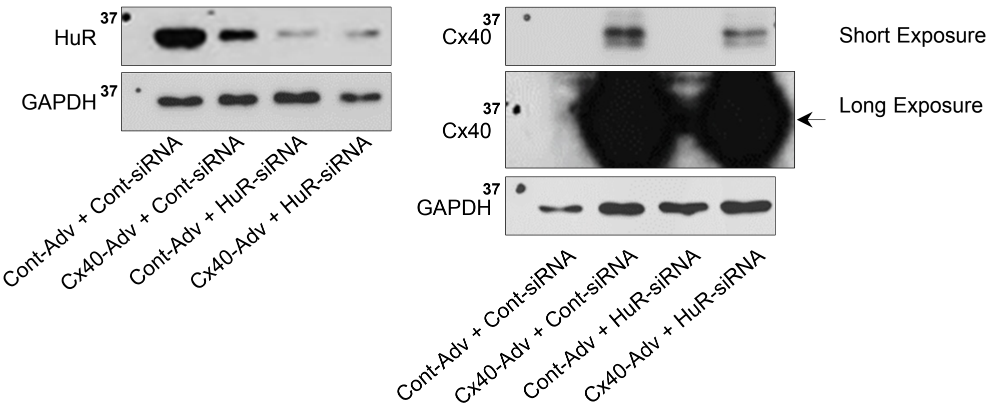

Figure S7. Representative Western blot image of HuR, Cx40, and GAPDH.

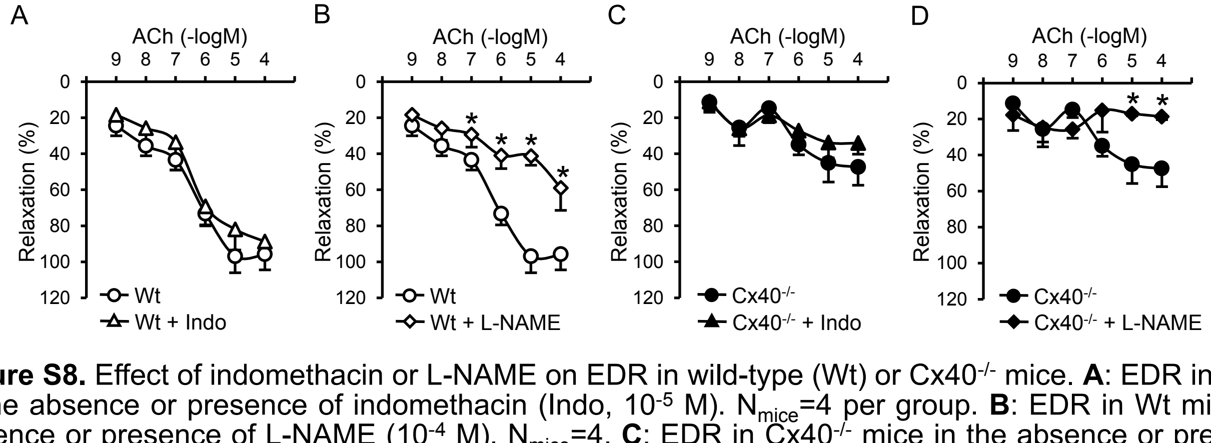

Figure S8. Effect of indomethacin or L-NAME on EDR in wild-type (Wt) or $Cx40^{-/-}$ mice. **A**: EDR in Wt mice in the absence or presence of indomethacin (Indo, 10^{-5} M). $N_{mice}=4$ per group. **B**: EDR in Wt mice in the absence or presence of L-NAME (10^{-4} M). $N_{mice}=4$. **C**: EDR in $Cx40^{-/-}$ mice in the absence or presence of indomethacin (Indo, 10^{-5} M). $N_{mice}=4$ per group. **D**: EDR in $Cx40^{-/-}$ mice in the absence or presence of L-NAME (10^{-4} M). $N_{mice}=4$. Data are mean ± SEM. **P*<0.05 vs. Wt or $Cx40^{-/-}$ without pretreatment. Statistical comparison between dose-response curves was made by two-way ANOVA with Bonferroni *post hoc* test.

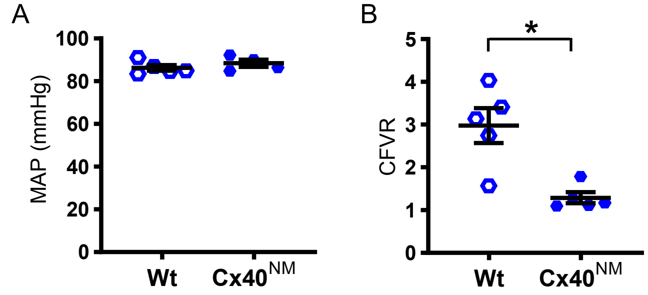

Figure S9. Mean arterial pressure (MAP) and coronary flow velocity reserve (CFVR) in Cx40 negative mutant (NM) knock-in ($Cx40^{NM}$) mice and age-matched wild-time (Wt mice). Mice carried with Tie2-driven Cx40 NM gene were obtained from Dr. Anthony W. Ashton (University of Sydney) and housed in our animal facility (*J. Am. Heart. Assoc.* 2020;9:e018327). **A**: MAP. Wt, n_{mice}=5; $Cx40^{NM}$, n_{mice}=4. **B**. CFVR. Wt, n_{mice}=5; $Cx40^{NM}$, n_{mice}=5. Data are mean ± SE. *P<0.05 vs. Wt. Unpaired Student's *t*-test (2-tailed) was used for comparisons of two experimental groups. In this study, we used both female and male mice to increase experimental number for the statistical analysis. There was no significant difference of MAP between male and female mice.

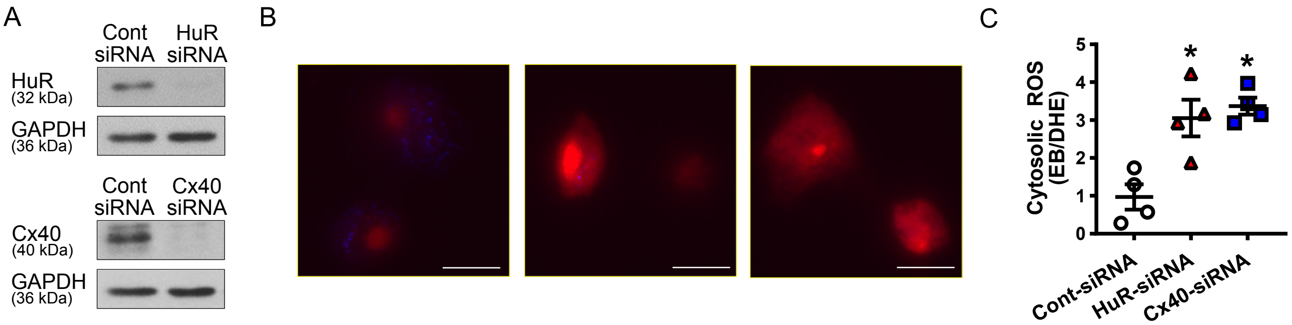

Figure S10. The deletion of HuR and Cx40 increases cytosolic ROS levels in human CECs. **A**. Representative western blot images of HuR, Cx40, and GAPDH. **B**. Representative photomicrographs of DHE staining. Bar=20μm. **C**. Summarized data shows cytosolic ROS levels in CECs after transfection with control-, HuR-, or Cx40-siRNA. $N_{experiment}$=4 per group. Over 30 cells were analyzed in each experiment. Data are mean ± SEM. *P<0.05 vs. Cont-siRNA. Statistical comparison among groups was made by one-way ANOVA with Bonferroni post hoc test.

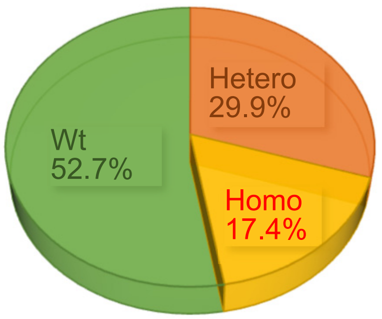

Figure S11. Proportional HuR genotype distributions in the litters from Tie2-$HuR^{+/-}$ parents. Genotype distributions within samples determined by genotyping are presented. The data are from 351 mice.

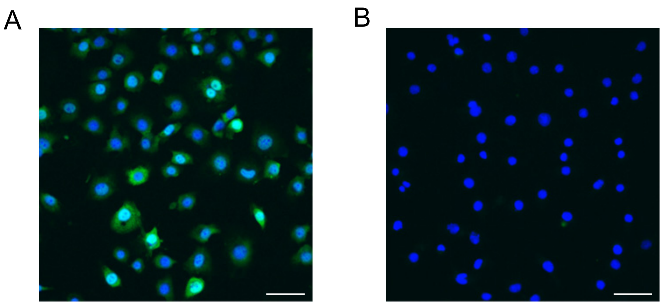

Figure S12. HuR staining in CECs. **A**: Representative photomicrographs showing CECs stained with anti mouse HuR antibody. **B**: Negative control images of CECs (without HuR antibody). Anti-mouse Alexa488 was used for the secondary antibody. HuR (green) and Hoechst (nuclear staining, blue). The image was taken by 20x objective lens. Bar=60 μm.

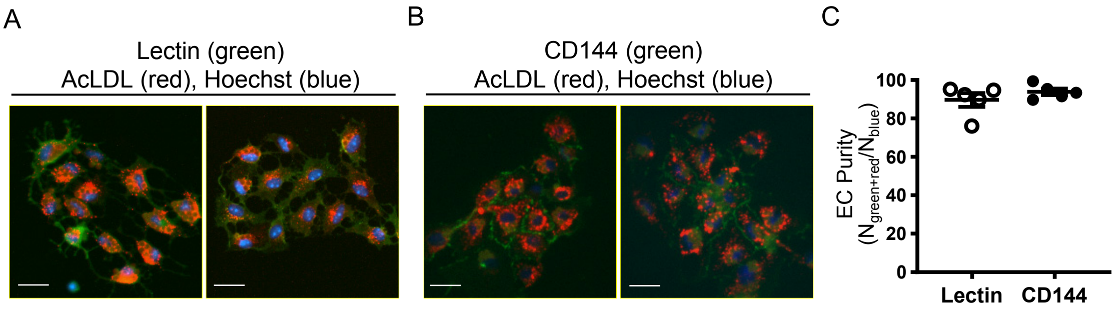

Figure S13. Purity test in mouse cardiac endothelial cells (CECs). **A-B**: Representative photomicrographs showing mouse CECs isolated by CD31-coated magnetic beads. After acLDL (red) treatment, CECs were stained with BS-lectin-FITC (Lectin, **A**) or CD144-Alexa488 (CD144, **B**). Nucleus was stained with Hoechst (blue). Bar=25μm. **C**: Averaged data showing the percentage of co-stained CECs with acLDL and Lectin or ac-LDL and CD144. There is no significant different of purity % between lectin- or CD144-stained cells. N_{mice}=5 per group. Five area per sample was randomly selected, and the averaged purity % per mouse was presented as a dot in the graph. Unpaired Student's *t*-test (2-tailed) was used for comparisons of two experimental groups.

Supplemental Tables

Table S1. Primers used for genotyping.

Strain	Gene	Forward	Reverse
Tie2-HuR$^{-/-}$	HuR	5'-GTTCCATGGCTCCCCATATC-3'	5'-AGCTTTGCAGATTCAACCTC-3'
	CRE	5'-ATTACCGGTCGATGCAACGAGT-3'	5'-CAGGTATCTCTGACCAGAGTCA-3'
Cx40$^{-/-}$	Wt	5'-TGGAGCCACAGTTGCAATGGT-3'	5'-TCTCTGACTCCGAAAGGCAAG-3'
	Cx40$^{-/-}$		5'-GCACGAGACTAGTGAGACGTG-3'
Cx40^{TG}	Cx40^{TG}-IRES	5'-CCAGGGCACCCTACTCAAC**A**-3'	5'-AGGGGCGGATCTCGAATCAA-3'
Cx40^{NM}	Cx40^{NM}-IRES	5'-CCAGGGCACCCTACTCAAC**G**-3'	5'-AGGGGCGGATCTCGAATCAA-3'

Table S2. Primers used for real-time PCR

	Forward	Reverse
Mouse HuR	GGATGACATTGGGAGAACGAAT	TGTCCTGCTACTTTATCCCGAA
Mouse Cx40	CCACAGTCATCGGCAAGGTC	CTGAATGGTATCGCACCGGAA
Mouse 18S ribosomal RNA	GTAACCCGTTGAACCCCATT	CCATCCAATCGGTAGTAGCG
Mouse GAPDH	TGACCTCAACTACATGGTCTACA	CTTCCCATTCTCGGCCTTG

Table S3. Gene list of Custom 384-well plate for Real time PCR.

Gene Symbol	Refseq	Catalog Number
Pecam1	NM_001032378	PPM03802C
Icam1	NM_010493	PPM03196A
Vcam1	NM_011693	PPM03208C
Flt1	NM_010228	PPM03066F
Kdr	NM_010612	PPM03057A
Flt4	NM_008029	PPM03068A
Vdac1	NM_011694	PPM04115D
Mcu	NM_001033259	PPM38489A
Micu1	NM_144822	PPM26993A
Micu2	NM_028643	PPM26935A
Mcur1	NM_001081059	PPM25703A
Smdt1	NM_026914	PPM35717A
Vegfa	NM_001025250	PPM03041F
Vegfb	NM_001185164	PPM03059A
Trp53	NM_001127233	PPM02931C
Mdm2	NM_010786	PPM02929C
Sp1	NM_013672	PPM04585F
Elavl1	NM_010485	PPM30921A
Hk1	NM_001146100	PPM05501A
Hk2	NM_013820	PPM03503F
Bcl2l1	NM_009743	PPM02920F
Bax	NM_007527	PPM02917E
Bak1	NM_007523	PPM03410F
Bcl2	NM_009741	PPM02918F
Hif1a	NM_010431, XM_006515477, XM_006515478	PPM03799C
Arnt	NM_001037737	PPM05265A
Epas1	NM_010137	PPM03309A
Arnt2	NM_007488	PPM03980D
Hif3a	NM_001162950	PPM05268B
Egln1	NM_053207	PPM31485B
Kcnn1	NM_032397	PPM04195A
Kcnn3	NM_080466	PPM04192A
Kcnn4	NM_001163510	PPM04199A
Trpc1	NM_011643	PPM31640A
Trpc4	NM_001253682	PPM04057A
Trpc6	NM_013838	PPM04056A
Mfn1	NM_024200	PPM37754A
Mfn2	NM_133201	PPM31777B
Opa1	NM_001199177	PPM36725E
Dnm1l	NM_001025947	PPM33638A
Fis1	NM_001163243	PPM26826A
Mgea5	NM_023799	PPM03692B
Ogt	NM_139144	PPM35599A
Stim1	NM_009287	PPM24556A
Stim2	NM_001081103	PPM36055A
Atp2a2	NM_001110140, NM_009722, NR_027838	LPM21786A
Atp2a3	NM_001163336	PPM04137A
Itpr1	NM_010585	PPM34085F
Gjb1	NM_008124	PPM04731A

Gja4	NM_008120	PPM26650A
Gja5	NM_008121	PPM37320A
Gja1	NM_010288	PPM05460A
Ptgs1	NM_008969	PPM03803F
Ptgs2	NM_011198	PPM03647E
Rac1	NM_009007	PPM03391F
Rac2	NM_009008	PPM03390A
Rhoa	NM_016802	PPM05485A
Cdc42	NM_001243769	PPM04527F
Pak1	NM_011035	PPM04553F
Pak2	NM_177326	PPM34220A
Nos2	NM_010927, XM_006532446	PPM02928B
Nos3	NM_008713	PPM03801A
Gch1	NM_008102	PPM25609F
Dhfr	NM_010049	PPM03698B
Ptgir	NM_008967	PPM04916A
Panx1	NM_019482	PPM31514B
Panx2	NM_001002005	PPM59262A
Panx3	NM_172454	PPM33577A
Iqgap1	NM_016721	PPM03344A
Edn1	NM_010104	PPM05274B
Ednra	NM_010332	PPM03063A
Ednrb	NM_001136061	PPM04840A
Sod1	M35725	PPM03582A
Sod2	NM_013671	PPM04371F
Sod3	NM_011435	PPM04365C
Cat	NM_009804	PPM04394C
Gpx1	NM_008160	PPM04345E
Nox1	NM_172203	PPM34199A
Cybb	NM_007807	PPM32951A
Nox4	NM_015760, NM_001285833, NM_001285835,	PPM27908A
Aggf1	NM_025630	PPM28417A
Ezh2	NM_001146689	PPM05645A
Wnk1	NM_001185020	PPM41181A
Wnk4	NM_175638	PPM26467A
Akt1	NM_001165894	PPM03377G
Akt2	NM_001110208	PPM03378C
Pten	NM_008960	PPM03379A
Mapk1	NM_001038663	PPM03571E
Mapk3	NM_011952	PPM03585E
Casp2	NM_007610	PPM02934C
Casp9	NM_015733	PPM03383F
Actb	NM_007393	PPM02945B
Gapdh	NM_008084	PPM02946E
GDC		PPM65836A
PPC		PPX63339
RTC		PPX63340

Endothelial function-focused real-time PCR plate was custom-made by QIAGEN Inc (SABIO Number CAPA38128-6:CLAM25240). One 384-well plate PCR array includes SYBR Green-optimized primers of quadruplicate 96 genes.

Table S4. Real time PCR result. Genes with altered mRNA levels in diabetic mice are listed.

Symbol	RefSeq Number	Description	Control		Diabetic		
			$2^{-\Delta\Delta CT}$	n	$2^{-\Delta\Delta CT}$	n	*p*-value
Vegfb	NM_001185164	vascular endothelial growth factor B	1.00 ± 0.04	6	0.71 ± 0.04	5	9.29E-04
Elavl1 (HuR)	NM_010485	ELAV (embryonic lethal, abnormal vision)-like 1 (Hu antigen R)	1.00 ± 0.02	6	0.76 ± 0.06	6	8.62E-03
Bcl2l1	NM_009743	BCL2-like 1	1.01 ± 0.07	6	1.66 ± 0.22	6	4.45E-02
Hif3a	NM_001162950	hypoxia inducible factor 3, alpha subunit	1.04 ± 0.12	6	1.96 ± 0.24	6	1.48E-02
Stim2	NM_001081103	stromal interaction molecule 2	1.00 ± 0.02	6	0.75 ± 0.07	5	2.86E-02
Atp2a2	NM_001110140 NM_009722 NR_027838	ATPase, Ca^{2+} transporting, cardiac muscle, slow twitch 2	1.01 ± 0.05	6	0.63 ± 0.12	5	4.19E-02
Gja5 (Cx40)	NM_008121	gap junction protein, alpha 5	1.01 ± 0.06	6	0.63 ± 0.01	5	1.54E-03
Rac2	NM_009008	RAS-related C3 botulinum substrate 2	1.14 ± 0.23	6	0.48 ± 0.10	5	4.79E-02
Edn1	NM_010104	endothelin 1	1.01 ± 0.05	6	1.59 ± 0.11	6	3.14E-03
Sod1	M35725	superoxide dismutase 1, soluble	1.01 ± 0.07	6	0.63 ± 0.02	5	2.84E-03
Nox4	NM_015760 NM_001285833 NM_001285835 XM_006508010 XM_006508012	NADPH oxidase 4	1.00 ± 0.03	6	0.60 ± 0.03	5	1.43E-05

Data are presented as mean ± S.E. Student's *t*-test was carried out to determine the significance between groups.

Table S5. Real time PCR result. Genes with altered mRNA levels in HuRKO mice are listed.

Symbol	RefSeq Number	Description	Wt		HuRKO		
			$2^{-\Delta\Delta CT}$	n	$2^{-\Delta\Delta CT}$	n	*p*-value
Icam1	NM_010493	intercellular adhesion molecule 1	1.04 ± 0.13	6	0.65 ± 0.07	6	2.50E-02
Mcur1	NM_001081059	mitochondrial calcium uniporter regulator 1	1.01 ± 0.05	6	0.82 ± 0.05	6	2.17E-02
Mdm2	NM_010786	transformed mouse 3T3 cell double minute 2	1.01 ± 0.05	6	0.68 ± 0.08	6	5.06E-03
Trpc1	NM_011643	transient receptor potential cation channel, subfamily C, member 1	1.01 ± 0.05	6	0.59 ± 0.08	6	1.90E-03
Trpc6	NM_013838	transient receptor potential cation channel, subfamily C, member 6	1.02 ± 0.10	5	0.52 ± 0.04	6	4.57E-03
Mfn1	NM_024200	mitofusin 1	1.01 ± 0.07	6	0.81 ± 0.03	6	2.18E-02
Opa1	NM_001199177	optic atrophy 1	1.01 ± 0.05	6	0.69 ± 0.07	6	3.08E-03
Dnm1l	NM_001025947	dynamin 1-like	1.01 ± 0.05	6	0.78 ± 0.07	6	1.72E-02
Gja5	NM_008121	gap junction protein, alpha 5	1.02 ± 0.09	6	0.41 ± 0.07	6	3.93E-04
Ednra	NM_010332	endothelin receptor type A	1.01 ± 0.05	6	0.75 ± 0.10	6	4.39E-02
Ednrb	NM_001136061	endothelin receptor type B	1.04 ± 0.13	6	0.71 ± 0.06	6	4.64E-02
Cybb	NM_007807	cytochrome b-245, beta polypeptide	1.03 ± 0.12	5	0.60 ± 0.11	5	2.74E-02
Nox4	NM_015760<br>NM_001285833<br>NM_001285835<br>XM_006508010<br>XM_006508012	NADPH oxidase 4	1.02 ± 0.08	6	0.70 ± 0.07	6	1.88E-02
Ezh2	NM_001146689	enhancer of zeste homolog 2 (Drosophila)	1.00 ± 0.04	6	0.51 ± 0.04	6	2.46E-06
Pten	NM_008960	phosphatase and tensin homolog	1.00 ± 0.02	6	0.75 ± 0.06	6	2.62E-03
Actb	NM_007393	actin, beta	1.01 ± 0.08	6	0.71 ± 0.08	6	1.94E-02

Data are presented as mean ± S.E. Student's *t*-test was carried out to determine the significance between groups. Note that the primer set for *Elavl1* (HuR) on the plate (Product # PPM30921A, QIAGEN) detects exon 5, not exon 2; therefore, real-time PCR was repeated using an exon-2-specific primer (Additional File 2: Table S2, reverse sequence in Exon 2) to confirm the deletion of HuR and the result is shown in Fig. 3C.

Materials

Antibodies

Target Antigen	Vendor or Source	Catalog #	Dilution Rate
Actin	Santa Cruz Biotechnology Inc (TX, USA)	sc-1616	1:4000
Alexa488 (anti mouse)	Thermo-Fisher Scientific, (MA, USA)	A-21200	1:2000
CD31	BD Biosciences (CA, USA)	553370	1:500
CD144	BD Biosciences (CA, USA)	53-1441	1:200
Cx40	Santa Cruz Biotechnology Inc (TX, USA)	sc-20466	1:4000
GAPDH	Thermo-Fisher Scientific, (MA, USA)	MA5-15738	1:5000
HuR	Santa Cruz Biotechnology Inc (TX, USA)	sc-5261	1:5000

siRNA

Target Genes	Vendor or Source	Catalog #	Working concentration
Control siRNA	Santa Cruz Biotechnology Inc (TX, USA)	sc-37007	100 nM
HuR siRNA	Santa Cruz Biotechnology Inc (TX, USA)	sc-35619	100 nM
Cx40 siRNA	Santa Cruz Biotechnology Inc (TX, USA)	sc-43078	100 nM

Cultured Cells

Name	Vendor or Source	Catalog #	Lot#	Sex (F, M)	Diagnosis
HCEC	Lonza (Basel, Switzerland)	cc-2585	305907	M	Control
HCEC	Lonza (Basel, Switzerland)	cc-2585	547320	M	Control
HCEC	Lonza (Basel, Switzerland)	cc-2585	7F4281	M	Control
HCEC	Cell Applications Inc. (CA, USA)	300-05a	2991	M	Control
HCEC	Lonza (Basel, Switzerland)	cc-2992	233691	M	Type 2 Diabetes
HCEC	Lonza (Basel, Switzerland)	cc-2992	234247	F	Type 2 Diabetes
HCEC	Lonza (Basel, Switzerland)	cc-2992	239103	F	Type 2 Diabetes
HCEC	Cell Applications Inc. (CA, USA)	300T2D-05a	3105	M	Type 2 Diabetes

Chemicals

Description	Source	Catalog #
Acetyholine	Sigma Aldrich, (MO, USA)	A9101
BS-I-FITC (lectin)	Sigma Aldrich (MO, USA)	L9381
Buprenorphine	Henry Schein(NY, USA)	42023017905
Collagenase II	Worthington Biochemical Corp. (NJ, USA)	LS004176
Diet (normal diet, 13% kcal from fat)	Lab Diet (MO, USA)	5001
Diet (high fat diet, 60% kcal from fat)	Envigo RMS Inc. (IN, USA)	TD.06414
dihydroethidium	Thermo-Fisher Scientific, (MA, USA)	D11347
Dil-acLDL	Thermo-Fisher Scientific, (MA, USA)	L3484
Dispase II	Worthington Biochemical Corp. (NJ, USA)	LS02109
DTT	VWR (PA, USA)	97061-340
Dynabeads ® Sheep Anti-Rat IgG	Thermo-Fisher Scientific, (MA, USA)	11035
Endothelial Cell Growth Supplement (ECGS)	Thermo-Fisher Scientific, (MA, USA)	356006
Fetal Bovine Serum (FBS)	Thermo-Fisher Scientific, (MA, USA)	MT35010CV
Heparin	Sigma Aldrich, (MO, USA)	H3149-100ku
HEPES	Sigma Aldrich, (MO, USA)	H4034-500G
Igepal	Sigma Aldrich, (MO, USA)	I8896-50ml
ImmunoCruz IP/WB Optima E (IP matrix)	Santa Cruz Biotechnology Inc (TX, USA)	sc-45042
indomethacin	Sigma Aldrich, (MO, USA)	I7378
In situ cell death detection kit	Roche (Basel, Switzerland)	11684795910
Iron-Supplemented Calf Serum	Thermo-Fisher Scientific, (MA, USA)	SH30072.04
Isoflurane	Henry Schein(NY, USA)	1169567762
KCl	Sigma Aldrich, (MO, USA)	P5405-250G
Ketamine	Henry Schein (NY, USA)	VINB-KET0-7021
Lipofectamin 3000 reagent	Thermo-Fisher Scientific, (MA, USA)	L3000008
L-NAME	Cayman Chemical (MI, USA)	80210
Matrigel	Thermo-Fisher Scientific, (MA, USA)	356237
Medium 199 (M199)	Thermo-Fisher Scientific, (MA, USA)	MT10060-CV

$MgCL_2$	Sigma Aldrich, (MO, USA)	M1028
miRNeasy Mini Kit	QIAGEN (CA, USA)	217004
$PGF_{2\alpha}$	Sigma Aldrich (MO, USA)	P0424
Phosphate Inhibitor Cocktail	Sigma Aldrich, (MO, USA)	P0044
Protease Inhibitor Cocktail	Sigma Aldrich, (MO, USA)	P8340
Rat anti-mouse CD31	BD Biosciences (CA, USA)	553370
RNase Free Water	Thermo-Fisher Scientific, (MA, USA)	MT-46-000-CI
RT^2 First Strand Kit	QIAGEN (CA, USA)	330404
Sodium Nitroprusside	Sigma Aldrich, (MO, USA)	71778
Streptomycin/penicillin	Thermo-Fisher Scientific, (MA, USA)	MT-30-002-CI
Streptozotocin (STZ)	VWR (PA, USA)	89149-800
Trypsin/EDTA	Thermo-Fisher Scientific, (MA, USA)	MT25052-CI
Xylazine	Henry Schein (NY, USA)	NADA # 139-236
Other general chemicals	Sigma Aldrich, (MO, USA)	
